# Evaluation of the iCARE Nigeria Pilot Intervention Using Social Media and Peer Navigation to Promote HIV Testing and Linkage to Care Among High-Risk Young Men: A Nonrandomized Controlled Trial

**DOI:** 10.1001/jamanetworkopen.2022.0148

**Published:** 2022-02-22

**Authors:** Robert Garofalo, Adedotun Adetunji, Lisa M. Kuhns, Olayinka Omigbodun, Amy K. Johnson, Kehinde Kuti, Olutosin A. Awolude, Baiba Berzins, Patrick Janulis, Ogochukwu Okonkwor, Bibilola Oladeji, Abigail L. Muldoon, Oluwaseun P. Amoo, Hannah Atunde, Bill Kapogiannis, Babafemi O. Taiwo

**Affiliations:** 1 Department of Pediatrics, Northwestern University Feinberg School of Medicine, Chicago, Illinois; 2 Department of Preventive Medicine, Northwestern University Feinberg School of Medicine, Chicago, Illinois; 3 Division of Adolescent and Young Adult Medicine, Ann & Robert H. Lurie Children’s Hospital of Chicago, Chicago, Illinois; 4 Department of Family Medicine, University College Hospital, Ibadan, Nigeria; 5 Centre for Child and Adolescent Mental Health, College of Medicine, University of Ibadan, Nigeria; 6 Department of Psychiatry, College of Medicine, University of Ibadan, Ibadan, Nigeria; 7 Department of Child and Adolescent Psychiatry, University College Hospital, Ibadan, Nigeria; 8 Department of Obstetrics and Gynecology, College of Medicine, University of Ibadan, Ibadan, Nigeria; 9 Infectious Disease Institute, College of Medicine, University of Ibadan, Ibadan, Nigeria; 10 Division of Infectious Diseases and Center for Global Health, Northwestern University, Chicago, Illinois; 11 Department of Medical Social Sciences, Northwestern University, Chicago, Illinois; 12 Eunice Kennedy Shriver National Institute of Child Health and Human Development, Bethesda, Maryland

## Abstract

**Importance:**

Nigeria has the fourth-largest HIV epidemic globally, yet high levels of social stigma inhibit HIV testing among Nigerian youths and young men who have sex with men (MSM).

**Objective:**

To report pilot data from iCARE Nigeria (Intensive Combination Approach to Roll Back the Epidemic in Nigerian Adolescents), a combination intervention using social media and peer navigation to promote HIV testing and linkage to care among high-risk youths and young men (hereinafter referred to as *young men*), including predominantly young MSM.

**Design, Setting, and Participants:**

This nonrandomized controlled study assessed an organizational and community-level 12-month, preintervention-postintervention pilot trial of a combination intervention designed to increase HIV testing uptake, increase the rate of identified seropositive cases, and improve linkage to care among young men, including MSM, using social media outreach and peer navigation. Data were collected from June 1, 2019, to May 30, 2020. Participants were young men aged 15 to 24 years in the city of Ibadan, Nigeria, and surrounding areas. Frequencies and percentages were examined, and a Fisher exact test was used to evaluate outcomes compared with historical surveillance data. Linkage to care was defined as 2 clinic visits, including HIV confirmation, within 2 months of a positive rapid test result.

**Intervention:**

Four peer navigators conducted social media outreach promoting sexual health and guiding individuals to HIV counseling and rapid testing in clinical, community, or home-based settings.

**Main Outcomes and Measures:**

Primary outcomes included the number of young men tested for HIV at university-based iCARE catchment clinics or by iCARE peer navigators in the community, the postintervention HIV seroprevalence of these groups, and linkage to care of participants diagnosed with HIV infection.

**Results:**

A total of 339 participants underwent testing for HIV (mean [SD] age, 21.7 [1.9] years), with 283 (83.5%) referred through social media. The main referral sources for social media were WhatsApp (124 [43.8%]), Facebook (101 [35.7%]), and Grindr (57 [20.1%]). Regarding testing location, participants chose home (134 [39.5%]), community-based (202 [59.6%]), or clinic (3 [0.9%]) settings. Eighty-six participants reported no prior HIV testing. Thirty-six participants (10.6%) were confirmed as HIV seropositive; among those, 18 (50.0%) reported negative test results within the past year, and 31 (86.1%) were linked to care. In two 6-month follow-up periods, the intervention increased HIV testing by 42% and 31%, respectively, and seroprevalence increased compared with historical trends with odds ratios of 3.37 (95% CI, 1.43-8.02; *P* = .002) and 2.74 (95% CI, 1.10-7.11; *P* = .02), respectively.

**Conclusions and Relevance:**

These findings suggest that use of iCARE Nigeria was associated with increased HIV testing and linkage to care in a high-risk, difficult-to-reach population, making it a promising combination intervention for young MSM.

**Trial Registration:**

isrctn.org Identifier: ISRCTN94590823

## Key Points

**Question:**

Does iCARE Nigeria, a combination intervention using social media and peer navigation, increase HIV testing and linkage to care among high-risk young men, including men who have sex with other men (MSM)?

**Findings:**

In this nonrandomized controlled trial that enrolled 339 youths and young men in Nigeria through social media and linked them to HIV testing and counseling, HIV testing increased by 31% to 42%, and seroprevalence increased compared with historical controls. Among 36 participants with positive test results for HIV, 31 (86%) were linked to care.

**Meaning:**

These findings suggest that use of iCARE Nigeria was associated with increased HIV testing and linkage to care in a high-risk, difficult-to-reach population that is critical to Nigeria’s efforts to control its HIV epidemic and holds promise in places where MSM sexual behavior and homosexuality are stigmatized.

## Teaser

This nonrandomized controlled trial reports 12-month findings from a combination intervention that uses social media outreach and peer navigation to promote HIV testing and facilitate linkage to care among youths and young men in Nigeria who are at high risk of HIV infection.

## Introduction

Nigeria has the highest HIV burden among countries in West and Central Africa and the fourth-largest HIV epidemic in the world.^1,2,3^ Access to HIV testing and treatment services is critical to help Nigeria achieve UNAIDS 90-90-90 goals to end this epidemic, defined as having 90% of all individuals with HIV knowing their diagnosis, 90% of those diagnosed with HIV infection receiving antiretroviral treatment, and 90% of those receiving treatment achieving suppression of their virus. To date, access to testing and treatment services has not been not evenly distributed across Nigerian demographic groups, with youth and men who have sex with men (MSM) among those with the greatest deficit.^1,2^

Approximately 20% of new HIV infections in Nigeria occur among youths and young men aged 15 to 24 years (hereinafter referred to as *young men*), yet only 17% are aware of their HIV status and only one-quarter have undergone testing for HIV.^1^ Men who have sex with men have the highest HIV prevalence in Nigeria (23%) and are the only group in whom prevalence continues to rise.^1,4^ Little has been published on HIV testing among young MSM in Nigeria.^5^ A stated goal of the revised Nigerian National HIV and AIDS Strategic Framework is to increase HIV testing among Nigerian youths, including young MSM.^6^

Among Nigerian MSM, factors limiting uptake of HIV testing are present at individual, network, and structural levels and include engagement in substance use and sexual behaviors associated with risk for HIV infection, social stigma, poverty, and the criminalization of same-sex relationships.^4,7,8,9^ In particular, the passage of the Same-Sex Marriage (Prohibition) Act of 2013 has led to increased violence against lesbian, gay, bisexual, and transgender (LGBT) communities and has restricted nongovernmental agencies from offering services such as HIV testing for gay men or other MSM.^10^ The criminalization of same-sex behavior in public and private spaces has made it more difficult to work with LGBT communities and has pushed young Nigerian MSM underground, limiting access to HIV prevention and treatment programs.^9,10^

Few interventions that promote HIV testing have been established for Nigerian youth, and to our knowledge, none have focused on young Nigerian MSM.^9,11,12,13,14,15^ Although these strategies have not been fully tested, a study^15^ found peer educators and/or social media to be effective in engaging Nigerian MSM in HIV testing. Similarly, although peer- and social media–based approaches have been used extensively to promote HIV testing among MSM in other parts of the world, few have targeted MSM in Africa, where two-thirds of countries criminalize same-sex relations and where access to testing and care are critical to eradicating this epidemic.^13,15,16^ In fact, a 2017 systematic review examining effectiveness of social media interventions to promote HIV testing, linkage to care, adherence, and retention among MSM^9^ identified 26 studies that met inclusion criteria; most were from high-income countries and none were from sub-Saharan Africa.

To address the urgent need for evidence-based, youth-targeted HIV interventions in sub-Saharan Africa, iCARE Nigeria (Intensive Combination Approach to Roll Back the Epidemic in Nigerian Adolescents) is a multiple-phase trial designed to adapt, investigate, and implement combination interventions to promote HIV testing and care outcomes among young men aged 15 to 24 years in Nigeria, focusing on young MSM. We report herein 12-month findings from the pilot phase of the iCARE combination intervention, which uses social media outreach and peer navigation to promote uptake of HIV testing and facilitate linkage to care.

## Methods

### Study Location

This study was conducted within HIV care programs of the Infectious Diseases Institute of the College of Medicine, University of Ibadan, and the University College Hospital, Ibadan, Nigeria. Both belong to the same university-based tertiary health care system and provide HIV tests at no cost. The Infectious Diseases Institute of the College of Medicine and University College Hospital are hereinafter referred to as *iCARE catchment clinics*. We selected Ibadan because it is the third-largest city in Nigeria and has fewer resources for key populations than other centers. The institutional review board of the University of Ibadan/University College Hospital Ethics Committee approved this study and waived written consent for the use of anonymized data.

### Overview of Study Design

The study was an organizational and community-level, 12-month, preintervention-postintervention nonrandomized controlled pilot trial of a combination intervention designed to increase HIV testing, increase the rate of identified seropositive cases, and improve linkage to care among young men living in Ibadan and the surrounding areas, including MSM, using social media outreach and peer navigation. We included all tested cases of HIV among young men aged 15 to 24 years at iCARE catchment clinics or via the iCARE intervention during the study period. Our control or comparison group included historical HIV testing surveillance data among all men aged 15 to 24 years at iCARE catchment clinics in the 6 months before intervention deployment and during the 12 months of intervention delivery. Although our social media outreach strategy specifically targeted young MSM, our primary outcome was deidentified HIV testing surveillance data for all men aged 15 to 24 years, as opposed to self-identified young MSM alone. We chose this approach because Nigerian HIV testing records do not contain valid data on same-sex behavior. Youths aged 15 years had to be emancipated to complete HIV testing and anonymous data collection in compliance with the 2014 Nigeria Federal Ministry of Health Guidelines for Young Persons’ Participation in Research and Access to Sexual and Reproductive Health Services.^17^ All study data were collected anonymously. The research conforms to the Transparent Reporting of Evaluations With Nonrandomized Designs (TREND) reporting guideline, and the trial protocol is provided in Supplement 1.

### Intervention

Community and stakeholder perspectives guided adaptation of evidence-based HIV testing, mHealth (defined by the National Institutes of Health as the use of mobile and wireless devices to improve health outcomes, health care services, and health research), and peer-navigation interventions for Nigerian youths.^18^ A manualized intervention specified staffing structure, general content of social media messages, and frequency of posts on 3 social media platforms used and trusted by Nigeria’s young MSM: WhatsApp, Facebook, and Grindr.

Four peer navigators received training based on Nigerian national guidelines and best practices for HIV testing among key populations, including an emphasis on human rights protections and maintaining safety and confidentiality. Additional training included (1) setting up social media profiles, (2) scripting social media messages, and (3) structuring interaction with participants (eg, how many times to send messages before moving on, providing responses as quickly as possible).

Trained peer navigators, male and female, operationalized the manualized intervention. Social media messaging focused on non-HIV health topics of interest as well as benefits of HIV testing, management of stigmatization of HIV testing, and peer-based social support. Depending on functionality of social media platforms, peer navigators’ activities included using existing or new closed groups on certain generic platforms or using geocoding capabilities with individual messaging on MSM-specific platforms. This created a sense of community related to iCARE Nigeria and focused on generating interest in HIV testing among young men.

For young men expressing interest in HIV testing, peer navigators conducted HIV rapid tests, offering options based on personal preferences and safety for clinic, community, or home-based testing. Young men with a positive result of a rapid test were referred and provided peer navigation to their preferred HIV treatment clinic for confirmatory testing and linkage to care. Those with negative HIV test results received reinforced HIV prevention education and were encouraged to test again 3 to 6 months after their initial test.

### Outcomes

Primary outcomes included (1) number of young men undergoing testing for HIV at university-based catchment clinics or by iCARE peer navigators in the community; (2) the postintervention HIV seropositivity rate of these groups; and (3) linkage to care of young men with a diagnosis of HIV infection. We defined linkage as 2 medical visits (inclusive of confirmatory testing) within 2 months of the positive HIV rapid test result. We used the Client Satisfaction Questionnaire to assess satisfaction with the intervention as a secondary outcome.^19^ We measured acceptability as a secondary outcome with a single item, “How much do you trust the results of today’s test?” (responses include to a great extent, somewhat, very little, and not at all).

### Data Collection

Data were collected from June 1, 2019, to May 30, 2020. For comparison purposes, surveillance HIV testing data were abstracted in deidentified and aggregate formats from clinic records for the 6-month period before the intervention and the 12 months of the intervention (in two 6-month periods). Abstraction included HIV tests of young men aged 15 to 24 years.

Youths undergoing testing for HIV by peer navigators as part of the iCARE intervention completed an optional questionnaire regarding demographic characteristics, HIV testing history, and intervention satisfaction. These data as well as subsequent data on linkage to HIV care among those with positive test results were captured in encounter forms using an anonymous identification number. Data were entered into REDCap, hosted at Northwestern University. This trial was retrospectively registered the year after study completion.

### Statistical Analysis

We used surveillance data to calculate the number of HIV tests performed and the seroprevalence rate during the intervention period at catchment clinics and tests performed by iCARE peer navigators outside these clinics (both initial and second tests). We compared the number of tests performed and seroprevalence rate in the 6 months before the intervention and the two 6-month periods after the intervention to determine the percentage increase in total tests attributable to the iCARE intervention. We used a χ^2^ test to examine overall change across periods, whereas Fisher exact tests were used to examine the statistical significance of change between specific periods. Descriptive statistics were used to evaluate intervention feasibility, acceptability, and satisfaction. We hypothesized that the iCARE intervention would have demonstrated preliminary efficacy and promise for advancing into a larger-scale effectiveness trial if (1) it increased the number of young men who undergo HIV testing through iCARE catchment clinics by at least 30% in each 6-month interval during the intervention period (to 12 months) and (2) it increased seropositive cases detected in this group by an odds ratio (OR) of 2.00. In addition, we expected to link at least 90% of new cases to HIV care. All analyses were conducted in R, version 4.0.4.^20^ Two-sided *P* < .05 indicated statistical significance.

## Results

**Figure 1.**
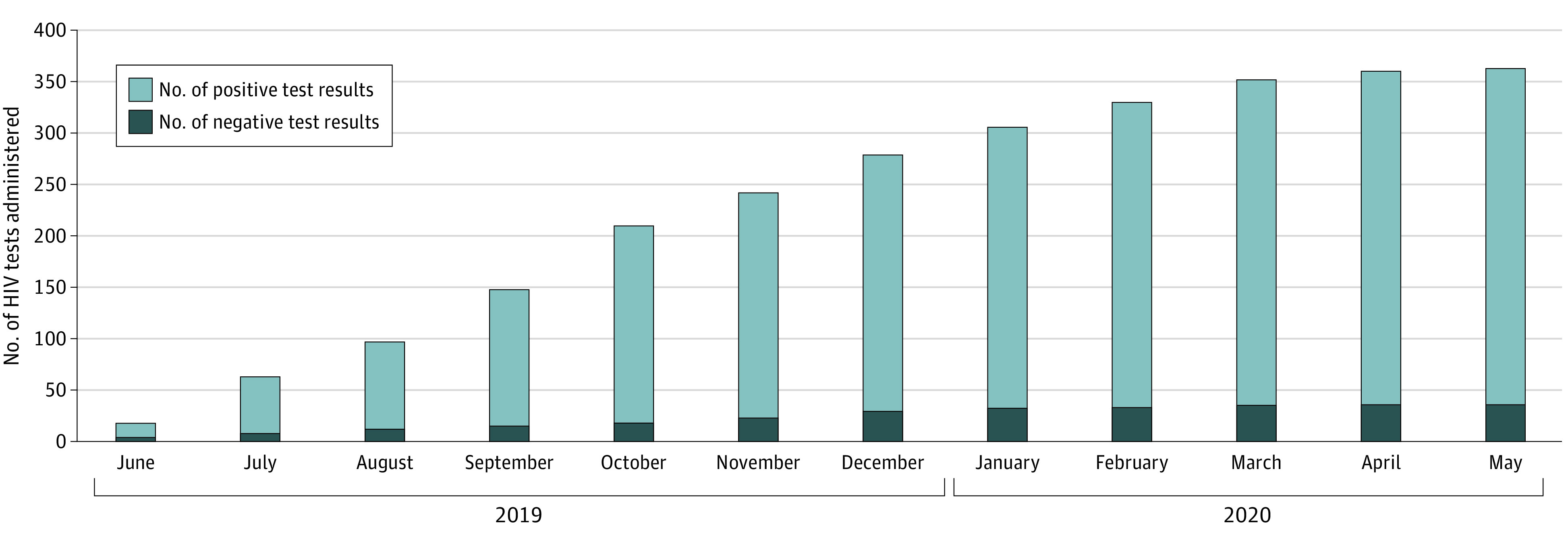
iCARE Nigeria Cumulative Number of HIV Tests and Seroprevalence Rate by Month (N = 363) iCARE Nigeria indicates Intensive Combination Approach to Roll Back the Epidemic in Nigerian Adolescents.

A total of 339 young men (mean [SD] age, 21.7 [1.9] years) underwent testing for HIV; of these, 24 received second tests at least 3 months after an initial negative test result. The mean (SD) number of tests per month was 30 (16). The cumulative number of tests with HIV seroprevalence by month during the entire 12-month period is depicted in Figure 1 (363 tests, including 339 initial tests and 24 second tests).

**Figure 2.**
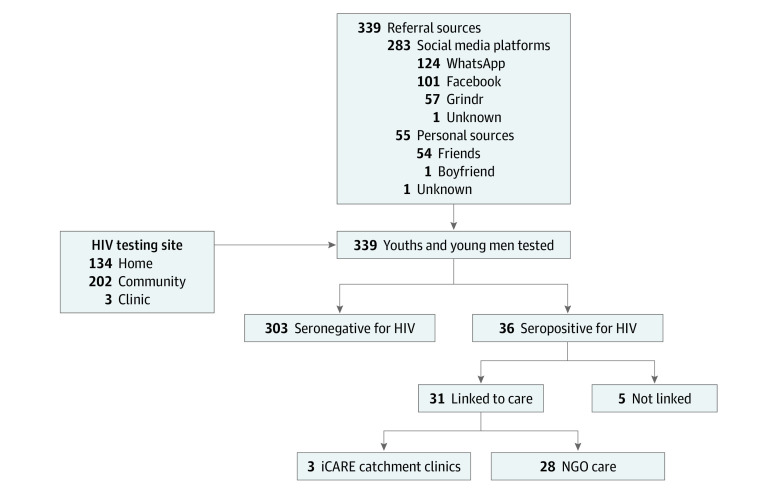
Trial Flow Diagram Data are from iCARE Nigeria (Intensive Combination Approach to Roll Back the Epidemic in Nigerian Adolescents).

Among individuals undergoing testing (N = 339), 283 (83.5%) were referred through social media and 54 (15.9%) by a friend. One young man (0.3%) was referred by a boyfriend/sex partner. Of those referred through social media, WhatsApp was the most popular platform (124 [43.8%]), followed by Facebook (101 [35.7%]) and Grindr (57 [20.1%]). For 1 participant referred by social media contact, no specific platform was documented. For 1 additional particpant, no original referral source was documented. Regarding testing location, 202 participants (59.6%) chose to test for HIV in a community-based setting, whereas 134 (39.5%) preferred and opted for home-based testing. Community-based testing locations emphasized privacy, including kiosks or private booths in local businesses such as restaurants or bars, parked vehicles, secluded areas of gardens and parks, or abandoned automated teller machine booths. Only 3 participants (0.9%) chose to test within a clinic setting. Eighty-six participants (25.4%) reported no history of HIV testing. Figure 2 is a flow diagram of our research design.

Thirty-six participants (10.6%) were confirmed to be HIV seropositive (35 on initial testing results and 1 on retesting result). Among those with positive test results for HIV, 18 (50.0%) reported a negative test result in the past year. Four HIV-seropositive individuals (11.1%) reported never having undergone previous testing for HIV.

All young men with a positive rapid test result completed a clinic visit to confirm their HIV status (1 participant had a negative test result at this visit and is thus counted among seronegative participants). Of these, 31 with confirmed HIV infection (86.1%) were linked to care. Another participant died and 4 were lost to follow-up.

HIV testing and seroprevalence data in the 6 months before intervention implementation and the two 6-month periods of the iCARE intervention are shown in the Table. During the 6 months before intervention implementation, a total of 423 HIV tests among young men aged 15 to 24 were conducted at the iCARE catchment clinics; 7 results were HIV positive, for an HIV prevalence of 1.7%. Among all young men undergoing testing during the first and second 6-month postintervention periods, tests conducted in the field by iCARE peer navigators led to a 42% and 31% increase, respectively, in HIV testing compared with the HIV testing reported previously in iCARE catchment clinics.

**Table.** HIV Prevalence Rates Among Young Men Aged 15-24 Years Before and After iCARE Nigeria Combination Intervention

Test location	Prevalence, No. HIV seropositive/No. tested (%)		
	6 mo before intervention	0-6 mo of iCARE	7-12 mo of iCARE
University-based iCARE catchment clinics^a^	7/423 (1.7)	7/328 (2.1)	5/272 (1.8)
iCARE outreach^b^	NA	23/242 (9.5)	13/121 (10.7)
All	7/423 (1.7)	30/570 (5.3)	18/393 (4.6)

Abbreviations: iCARE Nigeria, Intensive Combination Approach to Roll Back the Epidemic in Nigerian Adolescents; NA, not applicable.

^a^ Performed on site at Infectious Diseases Institute of the College of Medicine or University College Hospital.

^b^ Performed by an iCARE peer navigator in the participant’s home or a community setting after social media engagement.

HIV seroprevalence was higher among young men tested as part of the iCARE intervention compared with young men tested in iCARE catchment clinics before and after intervention (OR, 3.11 [95% CI, 1.38-7.08]; *P* = .002). Thus, the total HIV seroprevalence increased in each intervention period compared with the baseline pre-iCARE rate of 1.7% (7 of 423), to 5.3% (30 of 570) in the first period (OR, 3.37 [95% CI, 1.43- 8.02]; *P* = .002) and 4.6% (18 of 393) in the second period (OR, 2.74 [95% CI, 1.10-7.11]; *P* = .02). iCARE tests alone demonstrated a seroprevalence of 9.5% (23 of 242) and 10.7% (13 of 121) in each period, respectively. Seroprevalence did not significantly differ across the first and second intervention periods (OR, 0.81 [95% CI, 0.42-1.55]; *P* = .55).

In terms of linkage to care, 86.1% of cases had 2 visits with a clinician within 2 months of the positive HIV rapid test result. This rate was slightly lower than expected (90%).

Regarding the secondary outcomes of acceptability and satisfaction with the HIV testing process, participants in 340 of all testing events (93.7%) said they trusted the results of the test to a great extent. A total of 292 participants (80.4%) were very satisfied and 68 (18.7%) were satisfied. In addition, 358 participants undergoing testing (98.6%) would recommend iCARE to a friend.

## Discussion

In a 2019 systematic review and meta-analysis,^13^ HIV testing and engagement with the HIV treatment cascade among MSM in Africa was suboptimal and insufficient to achieve the UNAIDS 90-90-90 targets. Specifically, Stannah and et al^13^ reported that since 2011, only 50% of MSM in Africa reported being tested for HIV within the past 12 months. Much of the published literature on HIV testing and care engagement among MSM in sub-Saharan Africa focuses on surveillance and epidemiological factors or emphasizes individual-level risk factors (eg, sexual behaviors associated with a high risk of HIV infection). To date, limited prevention science has focused on the development of evidence-based interventions to increase HIV testing among adult or young MSM in sub-Saharan Africa.^21,22^ We report herein data on the first pilot HIV testing combination intervention targeting young men in Nigeria, including components of social media outreach and peer navigation focused on young MSM. Compared with historical data from the study site, a university-based HIV testing program in Ibadan, our approach was associated with an increased uptake of HIV testing among an often-hidden population of young men at very high risk of infection. Among all our participants, 25.4% reported no history of HIV testing, and 50.0% of those with positive HIV test results reported a negative test result within the past year. The intervention helped identify a substantially higher seroprevalence than was found in historical controls and then linked HIV-seropositive individuals with health care, despite multiple stigmas surrounding both HIV and same-sex behavior and sexual identity in Nigeria. The results of this study surpassed the thresholds for number of HIV tests and seroprevalence set in our hypotheses.

Data gaps and challenges to HIV research with MSM are largely the result of the stigmatization of MSM relationships and sexual behavior in many parts of the world.^13,23^ In countries such as Nigeria, where homosexuality is criminalized and where MSM often lead double lives—publicly engaging in heterosexual relationships and having same-sex relationships in secret—there is a high level of unmet need for HIV testing and improved access to MSM-friendly HIV prevention, care, and treatment services.^23^ For many Nigerian MSM, conflicts arise between cultural, religious, and family values and hidden sexual identities, challenging access to safe and confidential HIV testing and other preventive services.^24^ Historically, the burden of the provision of such services has fallen disproportionately on nongovernmental organizations (NGOs) with external funding sources (eg, World Health Organization) and with missions specific to caring for key populations at risk of HIV, such as LGBT-identified people or MSM.^25^ More traditional HIV health care service providers, such as university-affiliated tertiary health institutions, have rarely had a specific MSM focus, making the iCARE intervention within a university health system both innovative and of considerable public health significance. However, although iCARE staff and outreach activities were housed and based at the Infectious Diseases Institute of the College of Medicine, which is walking distance from University College Hospital, 336 of the young men in our study (99.1%) opted for and preferred community or home-based HIV testing rather than a clinic-based approach. Furthermore, only 3 participants who were seropositive for HIV (<10%) sought confirmatory testing and follow-up care at iCARE catchment clinics; most of them chose NGO-affiliated HIV care providers. This has implications for future research and programmatic efforts targeting MSM and speaks to a potential need to further cultivate partnerships between academic and university settings and NGOs to best reach and provide access to an often hidden and underground population such as young MSM. It also highlights the opportunity to strengthen MSM-friendly services in Nigerian tertiary health institutions. As part of the formative work related to the development and creation of the iCARE testing intervention, a great deal of effort was made to build community buy-in between the Infectious Diseases Institute of the College of Medicine and university health system and NGO stakeholders providing services to MSM in Ibadan.

After careful study design consideration, we chose to broadly define our target population as young men aged 15 to 24 years and limit data collection activities largely to surveillance data, given formative engagement with key youth and MSM stakeholders in Ibadan and prior literature suggesting limitations and challenges collecting self-reported information on sensitive and stigmatized topics such as one’s individual sexuality in Nigeria. However, our social media outreach strategies on generic platforms as well as MSM-specific platforms were almost entirely focused on reaching young MSM. Our outreach strategies differed by platform functionality. On the generic social media platforms, closed groups with upward of 200 to 250 members were created by participant referral, which leveraged the social networks of our MSM-identified peer navigators and other MSM participants. Peer navigators engaged young men—primarily MSM—within these closed groups with weekly posts or discussions on a range of general and sexual health topics, discussions of sexual orientation and gender identity and expression, topics related to stress and mental health, and safety tips for COVID-19, as well as more specific HIV-related posts on correct condom use, dispelling HIV transmission myths, preexposure prophylaxis, the importance of HIV testing, and early antiretroviral treatment as a prevention strategy. For MSM-specific platforms such as Grindr, which was the initial source of contact for 20.1% of our participants, peer navigators created iCARE Nigeria profiles with their own picture; profile content specifically referenced iCARE study activities and promoted HIV testing by encouraging people within a geographically close location to reach out for additional information. Feedback from peer navigators across all social media platforms as well as our high HIV seroprevalence of 10.6%, which is several-fold higher than the baseline seroprevalence among young men at iCARE catchment clinics, suggests the intervention reached our proposed target population of young MSM rather than simply a lower-risk general population of Nigerian young men in the desired age range.

### Limitations

This study has limitations that should be carefully considered within the context of the study design. Although this was a behavioral intervention, we approached data collection from a surveillance or a public health perspective. We chose this approach with an eye toward safety and potential for widespread scalability within a broader Nigerian public health framework, should the next phase of the larger multiple-site trial demonstrate effectiveness in reaching young men at risk and improving their HIV testing and care outcomes. Collection of such data would have limited our ability to engage young MSM, many of whom still live in fear. Our inference, therefore, is that prioritization of the confidentiality and safety of participants and staff in the study design directly contributed to the success of this study; however, this approach limited our ability to verify MSM status. The study had a relatively small sample size compared with large-scale surveillance on HIV testing. Additional limitations include those consistent with our nonrandomized design, including the preintervention-postintervention statistical analysis, concerns about possible confounders, and the fact that comparisons were not made and data were not collected looking at HIV testing targeting MSM that may be performed by local NGOs. In addition, generalizability is a concern because the study was conducted in a single geographic location and at 1 academic center. Finally, although we did not formally assess the impact of COVID-19 as part of our study procedures, there were notable changes (eg, decreases) in HIV testing numbers in our historical control surveillance data that may have been due in part to the social restrictions imposed during the last few months of study enrollment.

## Conclusions

Overall, these findings demonstrate acceptability of a combination intervention in reaching young men at risk, including young MSM, through social media and engaging them via peer navigation to promote HIV testing in Nigeria. These data suggest that this approach can be used successfully to identify new HIV cases in a population critical to Nigeria’s ongoing efforts to control the HIV epidemic and may hold promise throughout Nigeria as well as in other places where MSM behavior is stigmatized. To further investigate effectiveness and evaluate scalability, the second phase of iCARE Nigeria has been initiated, in which the combination intervention will be replicated at 5 sites across 3 Nigerian cities (Lagos, Jos, and Sagamu).

## Notes

Supplement 1.
Trial Protocol

Supplement 2.
Data Sharing Statement
