# Supplementary material for: Evaluation of the iCARE Nigeria Pilot Intervention Using Social Media and Peer Navigation to Promote HIV Testing and Linkage to Care Among High-Risk Young Men: A Nonrandomized Controlled Trial
Source: JAMA Netw Open. 2022 Feb 22;5(2):e220148. doi: 10.1001/jamanetworkopen.2022.0148 (PMC8864509; doi:10.1001/jamanetworkopen.2022.0148)
Supplement: Supplement 1. — Trial Protocol [file jamanetwopen-e220148-s001.pdf]

Version Information	Summary of Revisions Made	Rationale
Version		

Intensive Combination Approach to Rollback the Epidemic in Nigerian Adolescents: UG3 Phase

Protocol Number: iCARE Nigeria

National Clinical Trial (NCT) Identified Number: not applicable

Principal Investigators (PI):

Local PI: Olayinka Omigbodun, MBBS University of Ibadan

Collaborating PIs/Institutions:

Babafemi Taiwo, MBBS Northwestern University

Robert Garofalo, MD Lurie Children's Hospital

Sponsor: NIH/NICHD

Grant Title: Prevention and Treatment through a Comprehensive Care Continuum for HIV-affected Adolescents in Resource Constrained Settings (PATC3H) (UG3/UH3)

Grant Number*: 1UG3HD096920-01

Funded by: Eunice Kennedy Shriver National Institute of Child Health and Human Development

Version Number: 1.0

22 March 2019

Summary of Changes from Previous Version: *"Not applicable; this is the first version of the protocol."*

**Affected Section(s)**	**Summary of Revisions Made**	**Rationale**

CONFIDENTIALITY STATEMENT

This document is confidential communication. Acceptance of this document constitutes agreement by the recipient that no unpublished information contained herein will be published or disclosed without prior approval of the Principal Investigator or other participating study leadership and as consistent with the NIH terms of award.

Table of Contents

STATEMENT OF COMPLIANCE ... 1
INVESTIGATOR'S SIGNATURE ... 2
1 PROTOCOL SUMMARY ... 3
1.1 Synopsis ... 3
1.2 Schema ... 5
1.3 Schedule of Activities ... 5
2 INTRODUCTION ... 7
2.1 Study Rationale ... 7
2.2 Background ... 7
2.3 Risk/Benefit Assessment ... 10
2.3.1 Known Potential Risks ... 10
2.3.2 Known Potential Benefits ... 10
2.3.3 Assessment of Potential Risks and Benefits ... 10
3 OBJECTIVES AND ENDPOINTS ... 10
4 STUDY DESIGN ... 13
4.1 Overall Design ... 13
4.2 Scientific Rationale for Study Design ... 15
4.3 Justification for Intervention ... 15
4.4 End-of-Study Definition ... 15
5 STUDY POPULATION ... 16
5.1 Inclusion Criteria ... 16
5.2 Exclusion Criteria ... 16
5.3 Lifestyle Considerations ... 16
5.4 Screen Failures ... 16
5.5 Strategies for Recruitment and Retention ... 17
6 STUDY INTERVENTION(S) OR EXPERIMENTAL MANIPULATION(S) ... 17
6.1 Study Intervention(s) or Experimental Manipulation(s) Administration ... 17
6.2 Fidelity ... 19
6.2.1 Interventionist Training and Tracking ... 19
6.3 Measures to Minimize Bias: Randomization and Blinding ... 19
6.4 Study Intervention/Experimental Manipulation Adherence ... 19
6.5 Concomitant Therapy ... 19
7 STUDY INTERVENTION/EXPERIMENTAL MANIPULATION DISCONTINUATION AND PARTICIPANT DISCONTINUATION/WITHDRAWAL ... 19
7.1 Discontinuation of Study Intervention/Experimental Manipulation ... 19
7.2 Participant Discontinuation/Withdrawal from the Study ... 20
7.3 Lost to Follow-Up ... 20
8 STUDY ASSESSMENTS AND PROCEDURES ... 20
8.1 Endpoint and Other Non-Safety Assessments ... 20
8.2 Safety Assessments ... 22
Adverse Events and Serious Adverse Events ... 22
8.2.1 Definition of ADVERSE EVENTS AND Serious Adverse Events ... 22
8.2.2 Classification of an Adverse Event ... 23
8.2.3 Time Period and Frequency for Event Assessment and Follow-Up ... 23
8.2.4 Adverse Event Reporting ... 23

8.2.5 Serious Adverse Event Reporting 23
8.2.6 Reporting Events to Participants 23
8.2.7 Events of Special Interest 23
8.2.8 Reporting of Pregnancy 23
8.3 Unanticipated Problems 23
8.3.1 Definition of Unanticipated Problems 23
8.3.2 Unanticipated Problems Reporting 24
8.3.3 Reporting Unanticipated Problems to Participants 24
9 STATISTICAL CONSIDERATIONS 24
9.1 Statistical Hypotheses 24
9.2 Sample Size Determination 26
9.3 Populations for Analyses 26
9.4 Statistical Analyses 26
9.4.1 General Approach 26
9.4.2 Analysis of the Primary Endpoint(s) 26
9.4.3 Analysis of the Secondary Endpoint(s) 27
9.4.4 Safety Analyses 27
9.4.5 Baseline Descriptive Statistics 27
9.4.6 Planned Interim Analyses 27
9.4.7 Sub-Group Analyses 27
9.4.8 Tabulation of Individual Participant Data 27
9.4.9 Exploratory Analyses 27
10 SUPPORTING DOCUMENTATION AND OPERATIONAL CONSIDERATIONS 28
10.1 Regulatory, Ethical, and Study Oversight Considerations 28
10.1.1 Informed Consent Process 28
10.1.2 Study Discontinuation and Closure 29
10.1.3 Confidentiality and Privacy 29
10.1.4 Future Use of Stored Specimens and Data 29
10.1.5 Key Roles and Study Governance 29
10.1.6 Safety Oversight 29
10.1.7 Clinical Monitoring 30
10.1.8 Quality Assurance and Quality Control 30
10.1.9 Data Handling and Record Keeping 30
10.1.10 Protocol Deviations 31
10.1.11 Publication and Data Sharing Policy 31
10.1.12 Conflict of Interest Policy 31
10.2 Additional Considerations 31
10.3 Abbreviations and Special Terms 31
10.4 Protocol Amendment History 32
11 REFERENCES 32

STATEMENT OF COMPLIANCE

The trial will be carried out in accordance with International Council on Harmonisation Good Clinical Practice (ICH GCP) and the following:

- United States (US) Code of Federal Regulations (CFR) applicable to clinical studies (45 CFR Part 46, 21 CFR Part 50, 21 CFR Part 56, 21 CFR Part 312, and/or 21 CFR Part 812).

National Institutes of Health (NIH)-funded investigators and clinical trial site staff who are responsible for the conduct, management, or oversight of NIH-funded clinical trials have completed Human Subjects Protection and ICH GCP Training.

The protocol, informed consent form(s), recruitment materials, and all participant materials will be submitted to the IRB for review and approval. Approval of both the protocol and the consent form(s) must be obtained before any participant is consented. Any amendment to the protocol will require review and approval by the IRB before the changes are implemented to the study. All changes to the consent form(s) will be IRB approved; a determination will be made regarding whether a new consent needs to be obtained from participants who provided consent, using a previously approved consent form.

INVESTIGATOR'S SIGNATURE

The signature below constitutes the approval of this protocol and provides the necessary assurances that this study will be conducted according to all stipulations of the protocol, including all statements regarding confidentiality, and according to local legal and regulatory requirements and applicable US federal regulations and ICH guidelines.

Principal Investigator or Clinical Site Investigator:

Signed: ____________________ Date: ____________________

Name*:

Title*:

Investigator Contact Information

Affiliation*:

Address:

Telephone:

Email:

Signed: ____________________ Date: ____________________

Name:

Title:

Affiliation:

1 PROTOCOL SUMMARY

1.1 SYNOPSIS

Title: Intensive Combination Approach to Rollback the Epidemic in Nigerian Adolescents (iCARE Nigeria): UG3 Phase

Grant Number: UG3HD096920

Study Description: Two combination interventions, each with mHealth and Peer Navigation components, will be evaluated in two pilot studies among youth in Ibadan, Nigeria.

Pilot study 1 will evaluate the use of Social Media + Peer Navigation to improve HIV testing and linkage to care among Young Men Who Have Sex with Men (YMSM).

Pilot study 2 will evaluate Short Messaging System (SMS) + Peer Navigation to improve HIV Treatment Outcomes (Retention in Care, ART Adherence, Viral Suppression) among young People Living With HIV (PLWH) receiving care at the HIV Clinic, Infectious Disease Institute, of the College of Medicine, University of Ibadan/University College Hospital, Ibadan (IDI).

Objectives*: The primary objective of pilot study 1 (HIV Testing and Linkage to Care) is to determine the effect of Social Media Engagement + Peer Navigation on HIV testing and linkage to care among YMSM.

The primary objective of pilot study 2 (HIV Treatment Outcomes Intervention) is to determine the efficacy of the combination of SMS + Peer Navigation to improve HIV treatment outcomes (Retention in Care, ART Adherence, Viral Suppression) among youth with HIV receiving ART

Endpoints*: The primary endpoints for pilot study 1 are the number of young men who undergo HIV testing through IDI/UCH (i.e. on site at IDI/other locations within UCH, the University College Hospital, Ibadan, or by a peer navigator during community outreach) AND HIV incidence among the tested young men per IDI/UCH surveillance records at 48 weeks of the intervention in comparison to pre-intervention levels.

The primary endpoint for pilot study 2 is viral suppression, defined as viral load <200 copies/mL at Week 48 of the intervention.

Study Population:

The population for pilot study 1 is the total number of HIV tests completed, and resulting incidence, among youth in/around Ibadan.

The study population for pilot study 2 comprise youth living with HIV and receiving antiretroviral therapy (ART) at IDI.

Phase[*] or Stage: This is the UG3 (first; pilot) phase of the iCARE, Nigeria study

Description of Sites/Facilities Enrolling Participants: HIV Program at IDI is supported by the APIN Public Health Initiatives to provide ART to persons living with HIV (PLWH).

Description of Study Intervention/Experimental Manipulation: The combination intervention in each of the pilot studies includes peer navigation and mHealth components

Study Duration[*]: The study duration in pilot study 1 is 48 weeks after initiating the intervention, while in pilot study 2 the total study duration is 48 weeks after the last of 40 participants is enrolled, with each participant followed for 48 weeks.

1.2 SCHEMA

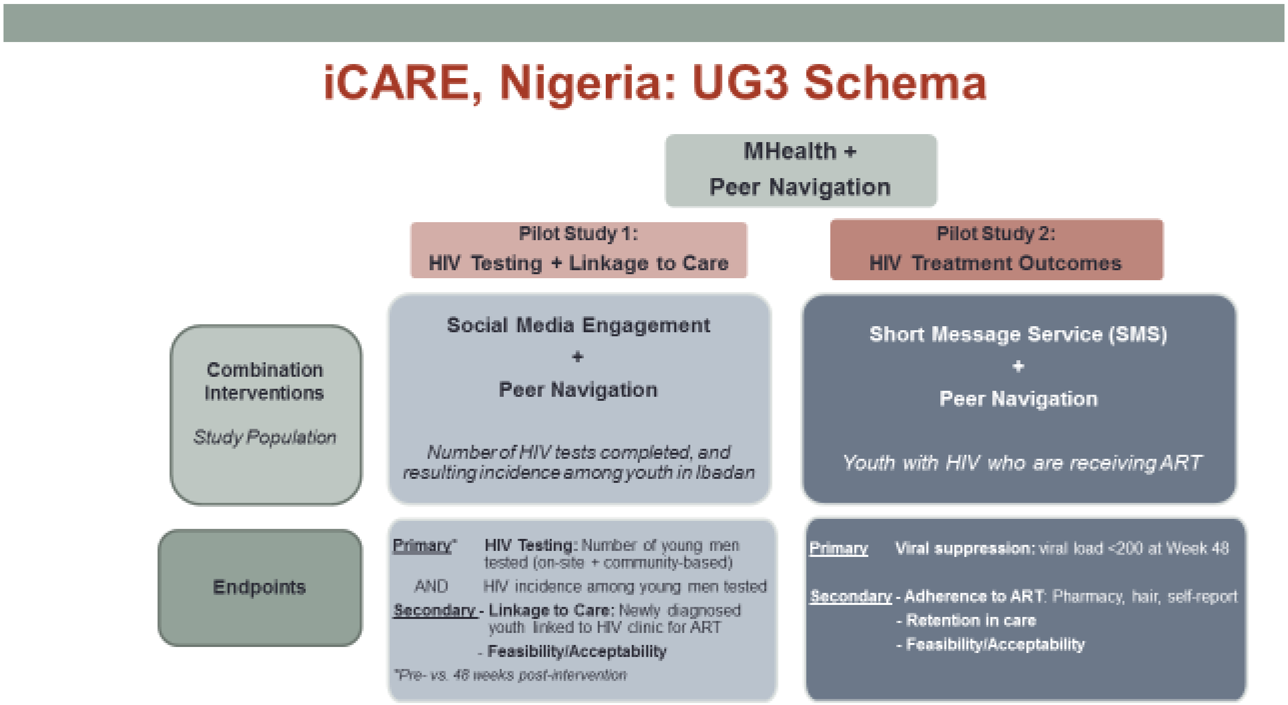

1.3 SCHEDULE OF ACTIVITIES

Pilot Study 1 (HIV Testing and Linkage to Care)

	Pre-intervention	Intervention Phase
Activities	• Collect IDI/UCH HIV testing seroincidence data for young men ages 15-24 for the 24 weeks preceding intervention	• Social Media Engagement<br>• Community Outreach and Peer Navigation including HIV Testing

Pilot Study 2 (HIV Treatment Outcomes)

	Baseline	Week 24	Week 48
Informed Consent	X		
Blood draw for Viral Load	X	X[a]	X
Adherence Assessment (Pharmacy Pick-up Records Abstraction, Self-Report,)	X	X	X
Hair sample for antiretroviral (ARV) drug concentration	X		X
Computer Assisted Personal Interviewing Questionnaires[b]	X	X	X
**SMS + Peer Navigation**	Baseline through week 48		
SMS Text Initiation	X		
Peer navigation Enrollment	X		
Intervention Feasibility, Acceptability and Satisfaction Questionnaires		X	X

[a] Per medical chart abstraction of standard-of-care viral load quantification.

[b]Computer assisted personal interviewing (CAPI): Demographic Characteristics, HIV Treatment Knowledge, HIV Medication Self-Efficacy, HIV Stigma, Substance Use, Depressive Symptoms

2 INTRODUCTION

2.1 STUDY RATIONALE

There is a need for effective interventions to improve HIV diagnosis and treatment among high risk youth in Nigeria.

2.2 BACKGROUND

There has been little progress in reversing the HIV epidemic in Nigerian youth aged 15-24 years. According to UNICEF,[1] Nigeria is one of the six countries with half of the global burden of adolescents (15-19 years) living with HIV. Youth in Nigeria have an approximately 50% higher prevalence of HIV compared to the general population.[2] Estimates are that 4.2% of youth have HIV, however, only 17% are aware of their HIV status.[3] New HIV infections have been on the decline in other age groups but not in youth. Recent estimates suggest that only 13% of youth ages 15-19 years and 25% of youth ages 20-24 years have ever been tested for HIV.[4] Since Nigeria has the highest number of perinatally HIV infected babies globally[5], the country's burden of youth living with HIV is expected to increase in coming years as these children age into adolescence.

Men who have sex with men (MSM) in Nigeria have the highest rate of HIV infection, with widespread discrimination and stigmatization driving the epidemic In fact, the prevalence of HIV infection among MSM is on the rise, despite reductions in other subgroups.[6] Among 862 MSM and transgender women (median age 24 years) screened for HIV in Lagos and Abuja from 2013-2016, the prevalence of HIV was 54.9% (66% in Abuja; 44% in Lagos).[7] Another study of 712 MSM in Abuja, Ibadan and Lagos, found HIV prevalence rates of 34.9%, 11.3%, and 15.2%, respectively.[8] Despite evidence of high rates of both HIV and condom-less sex (43% at last intercourse) among Nigerian MSM, estimates are that only 18% are reached by HIV prevention efforts and only 25% have tested for HIV in the last 12 months.[9] Clearly, young MSM (YMSM) in Nigeria have double demographic jeopardy with respect to HIV and should be the focus of urgent interventions to increase HIV testing and linkage to care. However, this challenge is daunting because MSM are extremely hard-to-reach and engage in Nigeria as a result of the Same-Sex Marriage Prohibition Act of 2014, which bans same-sex "amorous relationships" with penalties of up to 14 years in prison.

MSM often report sex with women. High rates of bisexual sex among Nigerian MSM suggest that interventions among MSM may directly impact on the broader epidemic. Among 557 MSM, approximately half also reported sex with women in the previous 2 months.[10] Consistent with this, a 2014 phylodynamic study estimated that almost 10% of new infections in females were attributable to epidemiological interactions with MSM.[11] High rates of transactional sex among MSM serve as another bridge to the general epidemic. A cross- sectional study of MSM in eight states in Nigeria found that 45% reported transactional sex, mostly with married men.[12] These data highlight the need to target MSM in outreach, testing and linkage efforts.

There are specific barriers to HIV testing and other desirable outcomes (linkage, retention, ART adherence) in the HIV continuum among youth, including YMSM. Although very little research has been done on HIV testing among youth in sub-Saharan Africa, a World Health Organization (WHO) study suggests that fear (e.g., of family/community reaction or life disruption) is a major barrier to HIV testing.[13] These fears are twice as high in YMSM.[13] In addition, evidence from South Africa suggests that another barrier is lack of youth-friendly testing services.[14] In a critical review of current global efforts to promote HIV testing in youth, Kurth and

colleagues recommended providing testing outside of traditional healthcare facilities (community-based, youth center, club, drop-in testing), making these services youth-friendly, and providing them for free or reduced cost.[15]

With regard to HIV care outcomes (e.g. retention, ART adherence), the developmental trajectories of youth are heterogeneous,[16] and cognitive development throughout adolescence and young adulthood is noteworthy for inconsistencies in the engagement of higher-order executive skills.[17] Therefore, mechanisms of support and intervention geared toward improving HIV care outcomes, must be sensitive to adolescent and young adult characteristics and priorities, including peer connection, sexual exploration, sensitivity to social rejection, diminished parental or adult influence, and variable self-regulatory capacity for challenging or emotionally sensitive tasks. Evidence also suggests that "simply forgetting" is the most cited barrier to medication adherence,[3] indicating this must be addressed in intervention efforts.

Combination interventions have been recommended by the WHO[18] as being most likely to have success for improving outcomes across the HIV continuum. A recent meta-analysis of 85 randomized controlled trials (14 from LMICs) of adult interventions to improve ART adherence and viral suppression, supports the superiority of combination versus single interventions,[19] noting additive effects. Many of the successful interventions combined short messaging service (SMS), social media, or peer-based support.[19]

Among the interventions tested to date, peer support has been shown to provide many benefits for adolescents across the HIV care continuum.[13] According to the WHO, peer navigation is well- suited to youth from a developmental perspective. Peers can be an important source of developmentally- grounded psychological support, helping to build confidence, resilience, reducing anxiety and promoting a sense of belonging.[13] Because of their common experiences, peers can help youth living with HIV to cope with fear, hopelessness, stigma and discrimination, and facilitate problem solving. Peer support can also help promote HIV testing or motivation and positive reinforcement to adhere to treatment.[13] Other potential benefits include assistance with disclosure, and help addressing mental health and substance use concerns that often negatively impact treatment outcomes.[20] For MSM, heightened stigma and rejection by family members and the larger community may undermine care engagement making peer support particularly helpful. Peer support may be also be pivotal for youth with perinatally acquired HIV, as caregivers withdraw support while they may be unprepared to shoulder the responsibility for their own HIV care.[21] Overall, peers may help youth navigate the sensitive and changing priorities of this stage of life, and help them overcome co-occurring challenges for this dynamic period of cognitive, behavioral, and social-emotional development.

In addition, **technology-based mHealth applications are an emerging area of behavioral intervention with evidence of efficacy across the HIV prevention and care continuum and potential for adaptation in the Nigerian context.** mHealth is a generic term that applies broadly to an increasingly large number of electronically delivered interventions and can include web-based tools like using social media outreach and SMS and e-mail messaging.[22] A review of 62 HIV-specific mHealth studies found that most target adults (45%) and featured alerts and reminders (60%) as a primary health promotion strategy.[29] Studies suggest positive effects across the HIV care continuum.[23,24] Interventions using mHealth approaches in sub-Saharan Africa have mostly focused on ART adherence with evidence of feasibility and efficacy. [25-28] Given prior success with mHealth strategies in other LMICs, adopting mHealth intervention approaches for Nigerian youth, including hard-to-reach YMSM, may be feasible, acceptable, efficacious, and scalable.

According to the Nigerian Communication Commission, Nigeria's teledensity, based on the numbers of active telephone subscribers, was 110% in 2017 with over 150 million telephone line subscriptions.[29] In addition as of June 2017, internet penetration in Nigeria was almost 50% with 92 million users, the highest in Africa.[30] In a 2015 cross-sectional study commissioned by Planned

Parenthood Global, among 1342 young persons in six Nigerian states (53% males and 46.9% females with mean age of 17 years) about 85% of those aged 15-24 years reported owning a mobile phone (unpublished data, Dr. A Olumide, The Adolescent Health Unit of the Institute of Child Health, University of Ibadan). Mobile phone access was similar in both rural and urban settings. Approximately 70% reported internet access. There are already social and support groups of Nigerian youth on both WhatsApp and Facebook, including YMSM. Independent bloggers (e.g., social, civil, entertainment) also have large followings in the hundreds to thousands.[36] mHealth approaches may be particularly effective to engage hard-to-reach MSM; recent evidence suggests that social media approaches are efficacious to increase HIV testing rates among MSM.[30] Social media is an internet-based platform that allows creation and exchange of user-generated content using mobile or web-based technologies, often with high user engagement.[31] Social media outreach has been suggested as one method to reach MSM, given widespread stigma and discrimination. In the US, "apps" are a key tool to access social support, meet friends, build community as well as for sexual networking among MSM [32]; anecdotal evidence suggests a similar trend in Nigeria. In a review of social media approaches to promote HIV testing globally (21 studies involved primarily MSM), social media was efficacious in increasing HIV testing (RR=1.50, 95% CI=1.28-1.76); effects increased for interactive versus passive approaches (RR=1.64, 95% CI=1.19-2.26),[31] particularly for those with peer-based interactions.[33,34] Participatory and peer-based interactions may enhance the importance and relevance of HIV-related social media activities, increasing motivation and support for completion. Cao and colleagues found that Facebook and Grindr were the most widely used platforms to promote HIV testing in high and middle-income countries (gay "chat" rooms and other gay-specific apps were also used);[31] however, these methods have not been fully tested in low income countries.

The interventions proposed in iCARE, Nigeria are evidence based. With respect to peer navigation, our group conducted one of the largest randomized trials of treatment supporters (among adults) in low-middle income countries at the Jos University Teaching Hospital,[35] which informs our peer-based approach. In the study (N=499) patient-selected treatment partners improved ART adherence and virologic success, though effects waned over time. Another study in Ibadan revealed the potential utility of mobile phones to improve adherence, though in a predominantly adult population.[36] The SMS component is based on Text Messaging Intervention to Improve Antiretroviral Adherence among HIV-Positive Youth (TXTXT)[37] which uses a two-way daily text reminder and response system designed by Garofalo and colleagues to improve ART adherence among 16-29 year-old U.S. adolescents and young adults. In comparison to the control condition, participants who received the TXTXT intervention were more than twice as likely to report >90% adherence to ART over the 6-month intervention period (OR = 2.12, 95 % CI 1.01–4.45, p<0.05), and the improvements were sustained at 6- months post intervention.[37] Both Health Resources and Services Administration (HRSA) and the Centers for Diseases Control (CDC) in the US have designated TXTXT as an effective HIV treatment intervention for ART adherence. While the original TXTXT intervention focused exclusively on ART adherence, for the purposes of the proposed study, we will adapt the intervention to deliver SMS text reminders not only for ART adherence, but also to focus on critical aspects of retention (e.g. clinic appointment and pharmacy refill reminders) as our primary outcome; viral suppression, is influenced by both. This intervention has not been adapted for use or tested in LMICs.

Community input is critical to develop effective, sustainable, culturally-competent interventions. To ensure that the interventions proposed in the iCARE, Nigeria study are targeted to youth and MSM in the local population and address the social context, we conducted a series of focus groups with youth living with HIV, HIV uninfected youth, YMSM, community based organizations, and other stakeholders in December, 2018. The results of the focus groups such as

the characteristics, ratio, and remuneration of the peer navigators were incorporated into the study design.

2.3 RISK/BENEFIT ASSESSMENT

2.3.1 KNOWN POTENTIAL RISKS

The primary risk to participation in this study is breach of confidentiality (related to being MSM identified or to HIV status). However, the risk to the participant is no greater than that encountered in standard counseling or HIV treatment services. The risk associated with the collection of blood from a vein in the arm (for viral load quantification), include pain or discomfort at the site of collection, temporary bruising at that site and very rarely, the site of blood collection may become infected or need medical treatment.

2.3.2 KNOWN POTENTIAL BENEFITS

The purpose of these pilot studies is to test two combination interventions among youths aged 15 to 24 years across the HIV care continuum in Nigeria. Equipoise is assumed, so there may be no benefit of participation in this study, however, participants may enjoy the opportunity to interact with the peer navigator and to receive text message reminders for medication and appointment adherence.

2.3.3 ASSESSMENT OF POTENTIAL RISKS AND BENEFITS

Protection Against Risks
For pilot 1, the HIV testing intervention, we will not enroll participants in research activities in order to reduce the potential risk of breach of confidentiality among YMSM given the criminalization of same-sex sexual behavior in Nigeria. In pilot 2, to protect the integrity of the youth's data, staff will assign each individual a unique patient identification number (PID) at study enrollment. This code number will be used for all study data. Only staff who have been trained in human subjects protection will have access to these PIDs.

Importance of the Knowledge to be Gained
Possible risks (i.e. potential confidentiality breeches) are outweighed by the new knowledge gained from testing this intervention among the study population, a population at very high risk of poor HIV outcomes.

3 OBJECTIVES AND ENDPOINTS

Pilot study 1: HIV Testing and Linkage to Care Intervention

OBJECTIVES	ENDPOINTS	JUSTIFICATION FOR ENDPOINTS	PUTATIVE MECHANISMS OF ACTION
Primary			
To determine the effect of	Number of young	While the focus of this	The

OBJECTIVES	ENDPOINTS	JUSTIFICATION FOR ENDPOINTS	PUTATIVE MECHANISMS OF ACTION
Social Media Engagement + Peer Navigation on HIV testing and linkage to care among young men.	men who undergo HIV testing through IDI/UCH (i.e. on site at ID/UCH or by a peer navigator during community outreach),<br>AND HIV incidence among the tested young men.	study is YMSM, the social conditions in Nigeria make it difficult to collect reliable data from this population. Data from young men in general will be collected to overcome this obstacle. An increase in HIV testing among male youth regardless of their sexual orientation is also desirable to stem the HIV epidemic	investigational intervention will alleviate barriers to HIV testing among youth, including YMSM
Secondary			
•To evaluate linkage of newly diagnosed youth to a clinic for ART<br><br>• To determine the feasibility, acceptability and satisfaction of the interventions	Linkage to care is a critical step in the HIV treatment continuum, and central to achieving Nigeria's HIV goals and the 90-90-90 declaration<br><br>a) Total number of peer navigator posts and respondent replies on social media platforms<br>b) Number of HIV testing encounters completed by peer navigator<br>c) Complaints or problems that result from social media interactions; and d) Results of the brief satisfaction questionnaire.	Ideally, all HIV diagnosed individuals would be linked to care; the public health goal is for 90% of HIV diagnosed individuals to be linked to care<br><br>The quantity and quality of interactions between peer navigators and at-risk youths, including their perception of the social media interface, are expected to impact the success of the interventions	Peer navigation will facilitate linkage to care<br><br>Feasibility and acceptability of the interventions will impact individual- and community-level adoption of the interventions, as well as the potential for scale up within and outside Nigeria
Tertiary/Exploratory			
Compare the HIV seroincidence data from this pilot study to data from other local HIV testing	Differences in HIV seroincidence between sites	There are multiple HIV testing agencies in Ibadan. Comparison of data between locations will	HIV testing outcomes are mediated by the methods

OBJECTIVES	ENDPOINTS	JUSTIFICATION FOR ENDPOINTS	PUTATIVE MECHANISMS OF ACTION
locations		provide some insight into the potential additive effect of the investigational interventions	used to reach and test the at-risk population

Pilot study 2: HIV Treatment Outcomes Intervention

OBJECTIVES	ENDPOINTS	JUSTIFICATION FOR ENDPOINTS	PUTATIVE MECHANISMS OF ACTION
Primary			
To determine the efficacy of the combination of SMS + Peer Navigation to improve HIV treatment outcomes (viral suppression, adherence to ART and retention in care) among youth receiving ART in IDI	Viral suppression, defined as viral load <200 copies/mL at Week 48 of the combination intervention	Viral suppression to levels below 200 copies/mL is a commonly used measure of successful ART. In addition, viral suppression is one of the indicators included in the UNIAIDS 90-90-90 declaration	The investigational intervention will improve adherence to ART which will in turn increase the odds of achieving and/or maintaining viral suppression.
Secondary			
• Determine the effect of the combination intervention on adherence to ART on youth receiving ART	ART adherence as measured by:<br>a) Pharmacy drug pick-up (% of days without medication)<br><br>b) Antiretroviral (ARV) drug concentration in hair<br><br>c) Self report	Pharmacy drug pick -up records and ARV drug concentration are objective measures of adherence while self-report is a complimentary subjective measure.	Practical assistance and other support provided by peer navigation combined with text message reminders and encouragement will mitigate the mediators of poor ART adherence among youth
• Determine the effect on retention in HIV Care	Retention defined as at least two care (non-study) visits in the preceding 6 months	Retention in care is surrogate marker of treatment success	The combination intervention will mitigate the mediators of poor ART adherence among youth
• Determine the feasibility, acceptability and satisfaction with the	a) Number of participants who request phones	Even if the proposed interventions have efficacy, their adoption will	Feasibility and acceptability of the interventions will impact

OBJECTIVES	ENDPOINTS	JUSTIFICATION FOR ENDPOINTS	PUTATIVE MECHANISMS OF ACTION
interventions	and number that report phone-related problems<br>b) Frequency of receipt of text messages as expected<br>c) Client Satisfaction Questionnaire	be impacted by issues such as access to telephones and acceptability of text messaging	individual- and community-level adoption of the interventions, as well as the potential for scale up within and outside Nigeria

4 STUDY DESIGN

4.1 OVERALL DESIGN

Two pre-post pilot studies will be conducted to investigate two interventions (each with mHealth + Peer Navigation components) that incorporate findings from focus groups conducted on youths living with HIV and other stakeholders in Ibadan in December 2018. Pilot study 1 will test a combination intervention that targets **HIV testing and linkage to care** while Pilot study 2 will test a combination intervention that targets **HIV treatment outcomes** (retention, adherence and viral suppression). These pilot studies will be implemented concurrently.

In Pilot study 1 (HIV Testing and Linkage to Care), we **hypothesize** that with the combination intervention of Social Media + Peer Navigation, i) the number of male youths who undergo HIV testing through IDI/UCH (i.e., at IDI/UCH or by peer navigators in the community) will be greater in the 48-weeks after the introduction of the intervention (in 24-week intervals) compared to 24 weeks before initiating the intervention, and that ii) the number of confirmed HIV positive cases divided by total number of tests (i.e., seroincidence) will be greater in the 48 weeks post intervention compared to pre-intervention (in 24-week intervals).

Trained peer navigators (including an Outreach Coordinator) will conduct HIV testing outreach to at-risk youth using social media platforms to promote HIV testing. For youths who express an interest in HIV testing, the peer will navigate them based on the youth's preference to any of the local HIV testing locations or the peer navigator will perform the HIV test wherever the participant feels most comfortable (e.g., home, bar or office). Youth who are navigated to HIV testing will be asked to complete an optional, brief, anonymous and self-reported questionnaire regarding their satisfaction with the intervention. Those diagnosed with HIV will be linked to their preferred HIV treatment clinic. While peer navigators will include youth men and women, the primary target of this study is YMSM.

The primary measures of the efficacy of the combination intervention for HIV testing/linkage are: a) number of male youth who undergo HIV testing through IDI/UCH (i.e. tested on site at IDI/UCH or by a peer navigator in the community) and b) the HIV seroincidence of this group, calculated as the number of confirmed HIV cases divided by the total number of tests. See definition of linkage to care in Section 8.1.

To determine efficacy of the combination intervention, HIV testing data in the 24 weeks before the intervention begins (weeks -24 to 0, i.e., pre-intervention) will be compared to the data at 24 and 48 weeks post- intervention. HIV testing data for all young men as opposed to YMSM alone is utilized mainly because HIV testing records do not routinely contain valid data on same-sex behavior.

As secondary objectives, we will assess, i) the proportion of newly diagnosed youth who are linked to care for ART, and ii) feasibility, acceptability, and satisfaction of the HIV testing/linkage combination intervention which will be assessed by: a) total number of peer navigator posts and respondent replies on social media platforms, b) number of HIV testing encounters by peer navigator, c) problems or complaints that result from social media interactions; and d) results of the brief anonymous satisfaction questionnaire.

Exploratory analyses will compare the HIV testing data obtained through the study to the data from other local HIV testing locations.

In Pilot study 2 (HIV Treatment Outcomes), the combination of Short Message Service (SMS; via adaptation of the TXTXT intervention) + Peer Navigation will be investigated to evaluate their effect on HIV treatment outcomes (viral suppression, ART adherence, and retention in care). We **hypothesize** that suppression (defined as < 200 copies/mL at 48 weeks) will be more likely post-intervention relative to pre-intervention.

We will enroll 40 youth (including virologically suppressed and viremic individuals) with HIV regardless of gender, ages 15-24 yrs who have been on ART for at least 3 months at the HIV clinic in IDI. To reflect the clinic population, enrollment will be monitored to ensure that no more than 35% are suppressed at study entry.

Trained peer navigators (one for 5 participants) will be selected from the IDI clinic using the following criteria: i) on ART, ii) virologically suppressed with viral load <200 copies/mL based on viral load in the preceding 12 months, if available, iii) 18 to 30 years of age, and iv) "doing well" according to their physician's subjective assessment. Each peer navigator will navigate 5 youths, by providing practical assistance and other support to optimize adherence. Provided support will be determined through a structured Needs Assessment process that will be conducted at the time of enrollment. In addition, peer navigators will foster a sense of community among the participants in their respective groups. Participants will receive SMS messages, adapted from the CDC recommended TXTXT, which includes daily, free, bi-directional and personalized text messages, delivered using a secure platform provided by Dimagi CommCare. The SMS will be personalized to remind and encourage the youth to adhere to their ART. Each participant will be followed for 48 weeks.

The primary efficacy measure for this combination intervention is the proportion of participants with viral load <200 copies/mL at week 48 of the combination intervention.

The secondary measures of efficacy are i) change in adherence (based on pharmacy pick up records, self-report, and hair antiretroviral drug levels) from baseline to weeks 24 and 48, ii) retention in care based on medical records, and iii) feasibility, acceptability and satisfaction.

Feasibility, acceptability, and satisfaction will be evaluated through a combination of logs maintained by peer navigators, self and interviewer-administered questionnaires. Exploratory assessments of factors associated with study outcomes will be performed using a socio-demographic and other surveys that will be conducted.

4.2 SCIENTIFIC RATIONALE FOR STUDY DESIGN

Evidence supports the combination of peer navigation and SMS reminders to improve HIV outcomes. In South African adults, Steward and colleagues used a cluster 3-arm randomized controlled trial (SOC/Standard of Care vs. SMS only vs. SMS + peer navigation) to evaluate the value of a combined intervention using peer navigation and SMS check-in messages in improving HIV treatment outcomes.[38] In the study, peers were trained to help other patients navigate HIV care and prevention through a minimum of monthly in-person meetings, phone and SMS contact as needed (minimum biweekly check-ins) and accompanying patients to clinic appointments as needed.[39,40] In addition to the SMS text check-in reminder with peer navigators, patients in the combination intervention arm also received SMS text “healthy living” reminders as well as reminders for clinic appointments. In comparison to the SOC arm, participants in SMS + peer navigation arm were almost twice as likely (OR 1.83, p<0.01) to be retained in care at 12 months (with retention defined for those on ART as at least 4 care visits within 12 months and for those not on ART defined as 2 or more visits in 12 months).[38] There was no significant difference in retention between the SMS alone vs. SOC arms. Consistent with this finding in adults, SMS alone failed to improve adherence to ART and co- trimoxazole prophylaxis in the only randomized trial of this intervention in youth\recently reported from Uganda.[41] These data suggest that combination interventions may be necessary in youth. Indeed, the combination of SMS text + peer navigation may be particularly suited to youth because it aligns with their developmental and social needs for peer engagement & acceptance and affinity for telecommunication, thus holding promise across the HIV care continuum.

4.3 JUSTIFICATION FOR INTERVENTION

Youth, particularly YMSM, are weak links in Nigeria’s response to the HIV epidemic. While there are many complicated factors that fuel the epidemic in these groups, a void of evidence-based interventions is remediable and necessary to advance the UNAIDS 90-90-90 goals. Therefore, we will investigate novel youth-specific approaches that include peer navigation and mHealth components and have been locally adapted using focus groups and stakeholder consultations.

4.4 END-OF-STUDY DEFINITION

These pilot studies are part of the first (UG3) phase of the iCARE, Nigeria study. If the hypotheses of this UG3 phase are met, randomized studies will be conducted in the second (UH3) phase.

5 STUDY POPULATION

The population for pilot study 1 is the total number of HIV tests completed, and resulting incidence, among youth in/around Ibadan. The study population for pilot study 2 comprise youth living with HIV and receiving antiretroviral therapy (ART) at IDI.

5.1 INCLUSION CRITERIA

Pilot study 1 (Testing and Linkage to Care)
HIV tests of young men ages 15-24 residing in Ibadan city and surrounding areas before (24 weeks) and during the intervention period (48 weeks) will be abstract from surveillance records for analysis.

NB: For the HIV testing and linkage intervention, no human subjects will be enrolled and no identifying data will be collected. Routine HIV testing data from surveillance records at IDI/UCH will be abstracted in a de-identified format. Intervention satisfaction data will be collected in anonymous format.

Pilot study 2 (Treatment Outcomes Intervention)
Inclusion criteria:

1. Age 15-24 years; if 15 years old must be emancipated i.e., has been granted status of adulthood by a court order, has lived independent of parental guidance for at least a year, is married, living on the street, or is head of a household
2. Living with HIV infection
3. Registered in the IDI clinic
4. On ART for at least 3 months. Participants may be virologically suppressed or viremic/failing on ART. We will monitor enrollment to ensure that no more than 35% of participants at baseline are virally suppressed (<200 copies/ml).
5. Understands and can read basic English and/or Yoruba
6. Willing and able to provide informed consent;
7. Intention to remain an IDI clinic patient during the 48-week follow-up period.

NB: There are no eligibility criteria specific to ART adherence or retention since these are subject to variation over time in youths, Cell phone ownership is not required; cell phones will be provided to participants without one.

5.2 EXCLUSION CRITERIA

Pilot study 2: Exclusion criteria

1. Inability to provide informed consent.
2. Youths who are 15 years old and not emancipated

5.3 LIFESTYLE CONSIDERATIONS

N/A

5.4 SCREEN FAILURES

Participants who do not meet the eligibility criteria will not be enrolled into the study.

5.5 STRATEGIES FOR RECRUITMENT AND RETENTION

The HIV testing and linkage pilot study will reach at-risk youth through social media and in-person outreach conducted by peer navigators, who will be trained to implement the manualized combination intervention, focused on promoting HIV testing and linkage to care.

To maximize random enrollment in the HIV treatment outcomes pilot study, a sampling frame will be created for eligible patients from medical records at IDI/UCH and stratified by status as virally suppressed versus viremic. A random sample will then be selected as every "n^th" participant based on the final ratio of target sample size to eligible patients in the sampling frame. As needed based on refusals or no-shows, the sampling frame will be re-constituted as a ratio of remaining sample needed to remaining eligible patients. These procedures will be followed until the accrual target of 40 is reached, while ensuring that no more than 35% have viral load < 200 copies/mL. In the event of a large number of refusals or no-shows that prohibits random sampling, the contingency plan will include convenience sampling of clinic patients approached at clinic visits.

A screening log will be maintained to track ineligible cases and reasons for ineligibility, as well as refusals and no-shows.

6 STUDY INTERVENTION(S) OR EXPERIMENTAL MANIPULATION(S)

6.1 STUDY INTERVENTION(S) OR EXPERIMENTAL MANIPULATION(S) ADMINISTRATION

Pilot study 1 (HIV testing and linkage to care Intervention) includes social media engagement and peer navigation.

Social media engagement: Trained peer navigators (male and female youth) will conduct outreach and engagement on social media platforms with peer navigators conducting the engagement on platforms that are used and trusted by Nigeria's YMSM. Peer navigator's activities will include using existing or creating new closed groups on these platforms, plus respondent-driven new invitations in order to generate a following. The peer navigators will receive a monthly stipend plus reimbursement for study-related expenses as incentive for their efforts.

Prior to deployment, the peer navigators will be trained on 1) profile set-up; 2) scripts for messaging, and instructions for structuring the interaction (e.g., how many times to message before moving on, providing responses as quickly as possible); 3) FAQs to guide unscripted interaction (e.g., Don't use your personal profile, Don't exchange personal information/photos; Do stick to pre-approved themes). The social media interactions will promote HIV testing.

Peer navigation: Peer navigators will also be trained to conduct community-based outreach and HIV testing, with emphasis placed on flexibility, building trust and maintaining confidentiality.[14,15,20,21] In addition, they will be trained in pre and post-test counseling and in the management of reactions to the test results. HIV testing will be promoted via social media and in-person interactions, e.g., at locations where young people may socialize locally. Social media messaging by peer navigators will focus on the benefits of HIV testing, management of stigmatization of HIV testing, and peer-based social support. The navigator will emphasize the advantages of HIV tests, e.g., "know your status," autonomy for self-management, benefits of early diagnosis and treatment.[13] In addition, the navigator will emphasize free testing and confidentiality of treatment.

For individuals who express interest in HIV testing, the peer will navigate them based on the youth's preference to either i) IDI/UCH testing site, ii) another testing site that the youth prefers, or iii) HIV test performed by the peer navigator at a location the youth finds comfortable. Youth with a positive HIV test will be navigated to the IDI/UCH clinic (or any other treatment site preferred by the youth) for ART initiation according to national guidelines. Youth who are navigated to HIV testing will be asked to complete an optional, brief, anonymous and self-reported questionnaire regarding their satisfaction with the intervention

Pilot study 2 <u>HIV treatment outcomes (retention, ART adherence and viral suppression) Intervention,</u> <u>also includes two components- peer navigation and SMS text messaging</u>

<u>Peer Navigation:</u> The peer navigators in this pilot study are defined as HIV positive, medication adherent role models living with a shared experience and a shared community membership as the populations they work with. The peer navigators will be selected from the IDI clinic using the following criteria: i) HIV-1 viral load < 200 copies/mL within the preceding 12 months (if available), ii) subjectively assessed as "doing well" by their physician, based on the categories- doing well, doing poorly, or variable, iii) willingness to volunteer as navigator, iv) successful completion of comprehensive training that includes modules on privacy and confidentiality. A designated clinic/study staff, assisted by the peer navigator, will conduct a Needs Assessment and ART education for participants. The navigator will initiate a minimum of two encounters per month (at least once every two weeks) with each of their assigned participants (in person or by texting/voice call/social media).[38,39]

Navigators are tasked with optimizing treatment outcomes by:

1) assessing and addressing barriers to care engagement (e.g., transportation to clinic);
2) providing referrals and linkage to supportive services;
3) attending appointments with participants as needed;
4) enabling early detection of those at risk of poor outcomes; and
5) fostering community and providing supportive care (e.g., support for disclosure of HIV status).

Each peer navigator will meet in person or by phone at least every two weeks with a designated clinic staff to discuss emerging issues with their assigned peers and formulate practical solutions including allocation of funds to participants based on the findings of the Needs Assessment.

Funds that are available to the clinic, and are not study-provided (e.g., through philanthropy or indigent fund), may be used to address some of the needs of study participants, based on availability. Peer navigators will receive a small stipend, which includes funds for their transportation/communication costs.

SMS text message reminders: This pilot study will utilize a locally adapted version of our CDC-recommended, TXTXT intervention.[37] Messaging will consist of daily, free, bi-directional and personalized text messages that will be delivered, using a platform provided by Dimagi CommConnect.[40] All text-related data will be securely stored by Dimagi per their privacy policy. The SMS messages will promote adherence and the content will be selected by and for the participant themselves with consideration given to each person's need for privacy and confidentiality and timed to coincide with individual dosing schedule.

Trained peer navigators will use a structured text message tailoring form to elicit message content and then set up automated messages in the Dimagi platform, i.e., set-up daily reminders according to the participants' dosing schedule and preferred message content. To protect privacy and confidentiality, we will encourage participants to delete text messages after taking medication, to use confidential messages that do not reveal HIV status or mention medications, and we will provide each

participant with a fact sheet about cell phone confidentiality (e.g., passcode protecting phone). Participants will have the option to choose a personalized message that may be changed as requested throughout the study period.

Bi-directionality is reflected in a series of follow-up messages. Participants will be asked to send a text message response indicating they have successfully taken their ART per schedule. An automated response system provides options for responding, including: 1) "Yes" or 2) "No." If the participant responds, "Yes" an affirmative and encouraging message will be sent in reply (e.g., "Well done!"); a "No" response will trigger an acknowledging and encouraging message (e.g., "You can do it!"). This 2-way message system provides a positive feedback to promote motivation, self-efficacy, and support.

Participants will use their own cell phones for receipt of messages, however, we will provide phones to those who do not have them (estimated to be <20% of clinic population) to ensure broad participation. Peer navigator will maintain logs of contacts with participants and phone-related problems

6.2 FIDELITY

Intervention fidelity will be determined by the proportion of intervention tasks completed, using a fidelity checklist.

6.2.1 INTERVENTIONIST TRAINING AND TRACKING

Peer navigators will undergo training prior to deployment. The training curriculum for research staff includes human subjects education, privacy and confidentiality, participant safety, and intervention and data collection training.

6.3 MEASURES TO MINIMIZE BIAS: RANDOMIZATION AND BLINDING

N/A

6.4 STUDY INTERVENTION/EXPERIMENTAL MANIPULATION ADHERENCE

N/A

6.5 CONCOMITANT THERAPY

N/A

7 STUDY INTERVENTION/EXPERIMENTAL MANIPULATION DISCONTINUATION AND PARTICIPANT DISCONTINUATION/WITHDRAWAL

7.1 DISCONTINUATION OF STUDY INTERVENTION/EXPERIMENTAL MANIPULATION

The study will terminate upon completion of the study procedures unless premature termination is mandated by the IRB or other constituted authority.

7.2 PARTICIPANT DISCONTINUATION/WITHDRAWAL FROM THE STUDY

Participants may withdraw or be discontinued from the treatment outcomes intervention study at any time for any of the following reasons:

i) Withdrawal of consent for any reason
ii) Potential for harm to the participant in the opinion of the site investigator or their physician

Peer navigators in either of the pilot studies will be discharged if there are concerns about their conduct on study, including violation of privacy and confidentiality requirements. Peer navigators who quit the study prematurely will be replaced.

7.3 LOST TO FOLLOW-UP

Participants will be considered lost to follow-up in Pilot Study 2 if they do not complete the week 48 visit. Measures to be taken by peer navigators to prevent loss to follow-up are similar to those already in place in the IDI/UCH clinic, including maintaining contact information up-to-date, using multiple methods of contact, and providing reminders for study visits.

8 STUDY ASSESSMENTS AND PROCEDURES

8.1 ENDPOINT AND OTHER NON-SAFETY ASSESSMENTS

Pilot study 1 (HIV Testing and Linkage to Care)

The primary endpoints for the combination intervention for HIV testing/linkage study are: a) number of male youth who undergo HIV testing through IDI (i.e. tested on site at the IDI/UCH or by a peer navigator during community outreach), and b) the HIV seroincidence of this group, calculated as the number of confirmed HIV cases divided by the total number of tests. To achieve this, HIV testing data for all young men, ages 15-24 will be abstracted from IDI/UCH surveillance records for the pre-post comparison (pre-post intervention at 24-month intervals).

Secondary endpoints include linkage to care of newly HIV diagnosed youth who undergo testing through IDI (i.e. tested on site at the IDI/UCH or by a peer navigator during community outreach). This is defined as registration in a clinic where ART is provided, and attendance at one follow up visit in that clinic within two months of registering. These data will be captured in HIV testing encounter forms used for surveillance purposes and tracked using an anonymous identification number.

We will also evaluate, as secondary objective, the feasibility and acceptability of the intervention via: a) total number of peer navigator posts and respondent replies on social media platforms, b) number of face-to-face interactions by peer navigators, c) complaints or problems that result from social media interactions; and d) results of a brief anonymous satisfaction questionnaire among those who complete HIV testing. To facilitate this, the outreach coordinator will de-brief with peer navigators regularly and maintain aggregate logs of social media platforms engaged, messages deployed, and responses. They will also catalogue HIV testing performed using encounter forms tracked with an anonymous identification number (NB: this anonymous identification number will facilitate linkage of HIV testing and linkage encounter data with satisfaction questionnaires).

The anonymous satisfaction questionnaire will be deployed via the secure data capture system, REDCap, hosted at Northwestern University. A remote link to the survey will be sent to those who complete HIV testing by a peer navigator. Aggregate social media tracking data and HIV testing encounter data will be entered into REDCap and stored for analysis.

Pilot study 2 (HIV Treatment Outcomes)

Study data will be collected at baseline (enrollment), Weeks 24 and 48 (See Schedule of Events, Section 1.3)

The primary endpoint for the HIV treatment intervention study is viral suppression defined as HIV < 200 copies/mL at week 48. We will collect blood via venipuncture for HIV viral load quantification at Entry and Week 48. Approximately 10 mL will be collected for this purpose and analyzed at the HIV Reference Laboratory, Virology Department, University of Ibadan, Nigeria.

Secondary endpoints are:

1) Adherence based on:
   - Hair ART levels (optional): Participants will be asked to have a small sample of hair (about 100 strands) cut from their head at Entry and Week 48. Collection of hair samples is non-invasive and does not require special equipment or storage. Hair analysis of uptake/adherence to ART will be completed at the University of California (UCSF) Hair Analysis Lab in batch. [42-45]
   - ART pharmacy pick-up: Pharmacy records of ART pick-up are captured as part of routine care at IDI and will be abstracted from the medical record.
   - Self-report of ART adherence via 30-day visual analogue scale (VAS) and 4-day missed doses. [46,47]

2) Retention in care

   This is defined as at least two care (non-study) visits to the IDI clinic in the preceding 6 months. Eligible visits include the bi-monthly "Youth Club" that takes place in the IDI clinic or any other non-study-mandated visit[38]

3) Feasibility, Acceptability and Satisfaction
   - Feasibility will be assessed using a number of metrics, including ratio of assigned participants to peer navigator (in comparison to plan), and total peer navigator activities and trends over time by type of activity. These activities will be recorded on peer navigation encounter forms. We will also track the number of participants who request phones, and all phone-related problems (e.g., lost phone access, cannot receive text reminders). An additional aspect of feasibility is intervention fidelity. Intervention fidelity will be determined by the proportion of intervention tasks completed, using a fidelity checklist.
   - To measure acceptability, we designed an acceptability questionnaire specifically for this study to include report of receipt of text messages and peer navigation services as expected, the degree to which services and messages are rated as intrusive/bothersome, and whether the services meet expectations for privacy.
   - To measure satisfaction, we will use an adapted version of the Client Satisfaction Questionnaire (CSQ-8) [54] including rating of the quality and quantity of service, outcome, and general satisfaction.

Additional psychosocial assessments that will be collected to describe the sample in detail include measures of HIV treatment knowledge,[48] HIV medication self-efficacy[49], HIV stigma,[50] depressive symptoms,[51] alcohol and drug use[52] and intervention satisfaction[53].

Biomarker data (i.e., viral load, hair analysis), pharmacy pick up, and retention in care data will be abstracted from lab reports or medical records and entered into REDCap for storage and analysis. Feasibility data will be collected and stored using administrative records and tracking logs. Self-reported adherence, acceptability and satisfaction, and additional psychosocial assessments will be collected using computer-assisted interviewer administered format.

8.2 SAFETY ASSESSMENTS

Participant safety will be monitored through weekly review of peer navigation activity logs and encounter forms and monthly tallies of completed satisfaction questionnaires. In addition, both study staff and peer navigators will be encouraged to report concerns about safety directly to the UI Principal Investigator (Prof. Olayinka Omigbodun). If any study staff discovers any untreated condition (e.g., onset of physical or mental health condition), they will refer the participant to appropriate treatment immediately. The other members of the core investigative team (Profs. Robert Garofalo and Babafemi Taiwo) will be informed of any significant safety concerns within 24 hours of the UI PI becoming aware of the event. Participant safety will be discussed at weekly and monthly research meetings, and a written log of all safety events will be maintained.

Since breach of confidentiality is a potential safety concern in these pilot studies, all study personnel and peer navigators will be trained regarding privacy and confidentiality. The training will include reviewing possible scenarios and key questions to assess risk. We will train staff to err on the side of caution and to contact the clinical supervisor or site investigator as needed. Under the guidance and direction of clinical supervisors (or site investigator), study staff will be trained when, if appropriate, to escort participants to the nearest emergency room or clinic in the event of an emergency.

ADVERSE EVENTS AND SERIOUS ADVERSE EVENTS

8.2.1 DEFINITION OF ADVERSE EVENTS AND SERIOUS ADVERSE EVENTS

While an adverse event (AE) is typically defined as any untoward medical occurrence in a participant enrolled in a study, for the purpose of these pilot studies in which no study drugs are provided or clinical care rendered, reportable AEs are limited to the events of special interest in section 8.2.7.

A serious adverse event (SAE) is one that meets one or more of the following criteria:

- Results in death
- Is life-threatening (places the subject at immediate risk of death from the event as it occurred)
- Results in inpatient hospitalization or prolongation of existing hospitalization
- Results in a persistent or significant disability or incapacity
- Results in a congenital anomaly or birth defect

- An important medical event that may not result in death, be life threatening, or require hospitalization may be considered an SAE when, based upon appropriate medical judgment, the event may jeopardize the participant and may require medical or surgical intervention to prevent one of the outcomes listed in this definition.

8.2.2 CLASSIFICATION OF AN ADVERSE EVENT

AEs and SAEs will be classified by the site investigator as related or not related to the study.

8.2.3 TIME PERIOD AND FREQUENCY FOR EVENT ASSESSMENT AND FOLLOW-UP

Unanticipated problems will be tracked in problem and adverse event logs throughout the study, and managed according to local practice

8.2.4 ADVERSE EVENT REPORTING

AEs, including SAEs, will be collated and presented as part of the study reports

8.2.5 SERIOUS ADVERSE EVENT REPORTING

All SAEs regardless of classification will be reported to the IRB and the NIH Program Officer within 3 working days of the study team becoming aware of its occurrence

8.2.6 REPORTING EVENTS TO PARTICIPANTS

In consultation with the IRB and the NIH Program Officer, the study team will notify participants and/or implement modifications to the study protocol

8.2.7 EVENTS OF SPECIAL INTEREST

Potential AEs include inadvertent disclosure of HIV status and significant negative emotional or social consequences of interactions and interventions that occur during the study. In addition, though unlikely based on findings of the recent focus groups and stakeholder consultations completed as part of the formative phase of this UG3 phase, there is a risk of arrest, incarceration or similar legal jeopardy as a result of the social and legal environment. These events will be reported as outlined in 8.2.1, 8.2.4 and 8.2.5

8.2.8 REPORTING OF PREGNANCY

Pregnancies that occur in the course of the study will be described in study reports. Since ART is not provided by the pilot studies, pregnancy outcomes will not be tracked by the study team.

8.3 UNANTICIPATED PROBLEMS

8.3.1 DEFINITION OF UNANTICIPATED PROBLEMS

Unanticipated problems involving risks to subjects or others include, in general, any incident, experience, or outcome that meets **all** of the following criteria:

- Unexpected in terms of nature, severity, or frequency given (a) the research procedures that are described in the protocol-related documents, such as the IRB-approved research protocol and informed consent document; and (b) the characteristics of the subject population being studied;
- Related or possibly related to participation in the research ("possibly related" means there is a reasonable possibility that the incident, experience, or outcome may have been caused by the procedures involved in the research); and
- Suggests that the research places subjects or others at a greater risk of harm (including physical, psychological, economic, or social harm) than was previously known or recognized.

8.3.2 UNANTICIPATED PROBLEMS REPORTING

All events that meet the criteria for an unanticipated problem will be reported to the IRB and the NIH Program Officer within 7 days of the study team becoming aware of its occurrence.

8.3.3 REPORTING UNANTICIPATED PROBLEMS TO PARTICIPANTS

In consultation with the IRB and Program Officer, the study team will implement participant notification and/or modifications to the study protocol

9 STATISTICAL CONSIDERATIONS

9.1 STATISTICAL HYPOTHESES

Pilot study 1: HIV testing and linkage to care

Primary Endpoints:

HIV Testing Hypothesis: The number of male youths who undergo HIV testing through IDI/UCH will be greater in the 48-weeks after the introduction of the intervention (in 24-week intervals) compared to 24 weeks before initiating the intervention.

Seroincidence Hypothesis: The number of confirmed HIV positive cases divided by total number of tests (i.e., seroincidence) will be greater in the 48 weeks post intervention compared to post intervention (in 24-week intervals).

Secondary Endpoints:

Linkage to Care Hypothesis: There will be a at least 90% linkage to care among those with newly diagnosed HIV

Feasibility Hypothesis: The combination intervention will be feasible
To assess feasibility and acceptability of the intervention, we will collate and describe: total number of peer navigator posts and respondent replies on social media platforms, b) number of face-to-face HIV testing encounters.

Acceptability and Satisfaction Hypothesis: The combination intervention will be acceptable to youths and they will be satisfied with the process
To assess acceptability of this intervention, we will monitor any complaints or problems that result from social media interactions; and d) results of the brief anonymous satisfaction questionnaire.

Exploratory Endpoint:

Seroincidence Hypothesis: Post-intervention seroincidence will be higher in the intervention site compared to other local HIV testing locations in Ibadan.

Pilot study 2 HIV treatment outcomes
Primary Endpoint:

Viral Suppression Hypothesis: Viral suppression (defined as < 200 copies/mL at 48 weeks) will be more likely post intervention relative to pre-intervention.

Secondary Endpoints:

Retention in Care Hypothesis: Retention in care will be greater post-intervention relative to pre-intervention. Retention in care is defined as at least two care (non-study) visits to the IDI clinic in the preceding 6 months. Eligible visits include the monthly “Youth Club” that takes place in the IDI clinic or any other non-study-mandated visit[38]

Adherence Hypothesis: Adherence will be greater post-intervention relative to pre-intervention. Adherence will be compared both for ART concentration, via hair sample, pharmacy pick-up records (100 minus percentage of days alive but without medication), and self-report (30-day visual analogue scale adherence, 4-day missed doses)

Feasibility Hypothesis: The combination intervention will be feasible
To evaluate the feasibility of the combination intervention for improving HIV outcomes, we will track the ratio of assigned participants to peer navigator (in comparison to plan) and the number of participants who request phones, and all phone-related problems (e.g., lost phone access, cannot receive text reminders). Intervention fidelity will be determined by the proportion of intervention tasks completed, using a fidelity checklist.

Acceptability and Satisfaction Hypothesis: The combination intervention will be acceptable to youths and they will be satisfied with the process
To measure acceptability, we will ask participants to self-report the frequency of receipt of text messages and peer navigation services as expected and the degree to which they find these services and messages intrusive/bothersome, and whether the services meet their expectations for privacy.

To measure satisfaction, we will use an adapted version of the Client Satisfaction Questionnaire (CSQ-8) [54] including rating of the quality and quantity of service, outcome, and general satisfaction.

Exploratory/Sensitivity Analyses: Proportion of participants with VL below the detection limit of the assay pre- versus post-intervention.

9.2 SAMPLE SIZE DETERMINATION

As a pilot study, this study is not calibrated to have adequate power to find statistical significance. Rather, we have pre-determined effect sizes satisfactory for future investigation as indicated in the analysis of primary endpoints section.

9.3 POPULATIONS FOR ANALYSES

The population for pilot study 1 is the total number of HIV tests completed, and resulting incidence, among youth in/around Ibadan. For pilot study 2, the population will include youth on ART at the IDI clinic.

9.4 STATISTICAL ANALYSES

9.4.1 GENERAL APPROACH

For descriptive statistics percentages will be presented for dichotomous outcomes (e.g., seroincidence) and means will be presented for continuous outcomes (e.g., adherence). Similarly, changes between pre and post-intervention periods will be summarized via odds ratios or mean differences. For each of the primary outcome measures, we will compare the pre-intervention period (i.e. 24 weeks before initiating intervention) versus post –intervention (i.e. 48 weeks after the introduction of the intervention, in 24-week intervals).

9.4.2 ANALYSIS OF THE PRIMARY ENDPOINT(S)

Pilot study 1
To examine changes in testing of male youth at IDI/UCH we will examine the number of male youths tested during pre- versus post-intervention. Again, we do not expect to have sufficient statistical power for statistical significance but expect an increase of ≥30% in total tests by male youth to be satisfactory. Similarly, we will estimate change in seroincidence by examining odds ratio of the contingency table of the proportion of male youth that test positive for HIV pre- versus post-intervention. An odds ratio of ≥2.0 pre- versus post-intervention will indicate be satisfactory evidence of the intervention on seroincidence.

Pilot study 2
To estimate the effect of the intervention on viral suppression, we will examine the odds ratio of the contingency table to compare the proportion of participants with viral load <200 copies/mL at study entry baseline) to the proportion at 48 weeks after the introduction of the intervention (in 24-week intervals). An odds ratio value ≥1.5 will be considered satisfactory evidence of efficacy of the intervention on viral suppression.

9.4.3 ANALYSIS OF THE SECONDARY ENDPOINT(S)

Pilot study 1

Linkage to Care: To estimate the effect of the intervention on linkage to care we will examine the odds ratio of the contingency table of the proportion of male youth that are linked to care pre versus post-intervention.

Feasibility and Acceptability: Frequency data will be used to provide descriptive estimates on all feasibility and acceptability outcomes.

Pilot study 2:

Retention in Care: To estimate the effect of intervention on retention in care we will examine the odds ratio of the contingency table of the proportion retained in care pre versus post-intervention.

Adherence: To estimate the effect of the intervention on adherence we will examine the mean difference in adherence pre versus post-intervention.

Feasibility, Acceptability and Satisfaction: Frequency data will be used to provide descriptive estimates on all feasibility and acceptability outcomes.

9.4.4 SAFETY ANALYSES

Safety issues that occur in the course of the studies will be described.

9.4.5 BASELINE DESCRIPTIVE STATISTICS

We will capture age, gender, and mode of infection (e.g., horizontal versus vertical) for descriptive purposes, as well as ethnicity/tribal affiliation, religion, employment, highest level of education, and marital/partnership status. These variables will be measured through a combination of self and interviewer administered survey deployment at baseline.

9.4.6 PLANNED INTERIM ANALYSES

N/A.

9.4.7 SUB-GROUP ANALYSES

We do not plan to perform subgroup analysis for the pilot phase.

9.4.8 TABULATION OF INDIVIDUAL PARTICIPANT DATA

N/A

9.4.9 EXPLORATORY ANALYSES

We will compare the HIV seroincidence data from this pilot study to data from other local HIV testing locations to examine if these proportions differ.

10 SUPPORTING DOCUMENTATION AND OPERATIONAL CONSIDERATIONS

10.1 REGULATORY, ETHICAL, AND STUDY OVERSIGHT CONSIDERATIONS

10.1.1 INFORMED CONSENT PROCESS

In pilot study 1, while no individuals will be enrolled for the purposes of assessment of primary outcomes, individuals who complete HIV testing will be consented prior to completion of the satisfaction survey (via a consent statement with waiver of documentation of consent). For human subjects research in pilot study 2, no research data will be collected prior to informed consent. Consent is conducted in a private room, by trained research staff. The consenting process will ensure autonomy of the youth by explicitly stating that participation in the research is voluntary, and that the youth is free to participate or withdraw at any time. The consenting procedure, among other elements, will cover: a) sources of potential harm (e.g., inadvertent disclosure even if the possibility is remote, or routine risks such as from blood draw); and b) importance of the knowledge to be gained (e.g. findings may improve understanding of how to improve the health for youths living with HIV)

While telephones will be provided to a small number of youths who do not have one and a small sum will be made available to facilitate provision of practical support (based on needs assessment) in pilot study 2, there will no overt or covert coercion, enticement or intimidation of the participants.

10.1.1.1 CONSENT/ASSENT AND OTHER INFORMATIONAL DOCUMENTS PROVIDED TO PARTICIPANTS

Determining Capacity. The cognitive capacity of youths will be carefully assessed as part of the consenting process. The trained Study Coordinator will proceed through each component of the consent, written to Nigerian primary school or below reading level, and will make an assessment of the youth's decisional capacity to consent prior to signing. Potential participants will be asked questions designed to assess their capacity to understand what the study is about, appreciate, reason with, and express a choice about participation in the protocol.

Parental consent

Parental consent is not required to participate in Pilot Study 2. In compliance with the 2014 Federal Ministry of Health Guidelines for Young Persons' Participation in Research and Access to Sexual and Reproductive Health Services in Nigeria, youths 16 years and older or emancipated minors aged 15 years are exempt from parental consent. This guidance defines an emancipated minor as a person under the age of 18 years who has been granted status of adulthood by a court order, has lived independent of parental guidance of at least a year, is married, living on the street, or is head of a household.

Parental consent may decrease participation rates because some youth will fear that their HIV status may be "outed" as a result of participation. Furthermore, the nature and scope of the proposed research do not pose more than "minimal risk" to participants To compensate for waiver of parental

consent, participants receive a formal individual assessment of capacity to consent (above) to ensure their understanding of study goals, procedures, and risks from disclosure of sensitive information.

10.1.1.2 CONSENT PROCEDURES AND DOCUMENTATION

Informed consent as described above must be obtained and documented before any human subjects research. This applies to completion of the consent statement in pilot 1 prior to the satisfaction questionnaire and pilot study 2 (HIV Treatment Outcomes Intervention).

10.1.2 STUDY DISCONTINUATION AND CLOSURE

While the pilot studies will run concurrently, it is anticipated that pilot study 1 will end before pilot study 2 due to the fixed period of observation and abstraction of surveillance data in pilot 1 and the need for individual-level follow up beyond the accrual period in pilot 2.

10.1.3 CONFIDENTIALITY AND PRIVACY

To protect the integrity of the participant's data, staff will assign each individual a unique participant identification number (PID) at study enrollment. This code number will be used for all study data. We will maintain a list of participants with links between identifying information and code numbers to avoid any duplication in cases and to facilitate follow-up in pilot 2. Only staff who have been trained in human subjects protection will have access to these lists, which will be kept on a secure server with password protected access. Consents are stored in a locked cabinet away from workstations and are only accessed in the event of a consent amendment or an audit. Locator files are kept in a locked cabinet in the research area, separate from data files, and are updated at each research visit.

10.1.4 FUTURE USE OF STORED SPECIMENS AND DATA

N/A

10.1.5 KEY ROLES AND STUDY GOVERNANCE

**Principal Investigator**
Olayninka Omigbodun, MBBS
University of Ibadan/University College Hospital
Address Ibadan, Nigeria
Phone Number +234=813-224-3158
Email olayinka.omigbodun@gmail.com

Prof. Omigbodun is the site PI at University of Ibadan and will oversee all study activities.

10.1.6 SAFETY OVERSIGHT

Oversight will be obtained through study-specified notification of both the IRB and the Program Officer if an SAE and unanticipated event occurs. In addition, a Data Safety Monitoring Board will perform

periodic review of safety data and study progress. This evaluation will occur 24 weeks after initiating pilot study 1 and after the first 20 participants reach week 24 in pilot study 2. Subsequent evaluations will occur annually thereafter for the duration of the study. An evaluation can be requested by the study at any time if there are concerns about participants' safety in the opinion of the investigators. The study statistician will provide a report of the following parameters to the study team for review at each evaluation:

Assessment of study progress (including actual vs. expected accrual, participant losses),
Timeliness and quality of data submission.

And the following efficacy and safety data for each study:
Pilot study 1 (Testing and Linkage to Care)

1. Number of HIV tests performed and seroincidence
2. Summary of the peer navigator logs that relate to safety of at-risk youth on social media and in outreach activities
3. All AEs and SAEs regardless of classification
4. Summary of the Feasibility and Acceptability results

Pilot study 2 (HIV Treatment Outcomes)

1. Enrollment data
2. Available viral load results
3. All AEs and SAEs regardless of classification
4. Summary of results from questionnaires and surveys that relate to safety of participants

10.1.7 CLINICAL MONITORING

Clinical monitoring will occur according to the local standard of care

10.1.8 QUALITY ASSURANCE AND QUALITY CONTROL

To ensure accuracy of collected data, a monthly data audit will be conducted

10.1.9 DATA HANDLING AND RECORD KEEPING

10.1.9.1 DATA COLLECTION AND MANAGEMENT RESPONSIBILITIES

We have developed systematic protocols for data handling and storage over multiple cohort studies. We maintain both paper files and computer files for each participant. Paper files include: 1) locator information, 2) informed consent, 3) any paper data forms. The first two have identifying information, and are linked to the data by the patient identification number (PID).

Computer files consist of the tracking data base, and study data files. Tracking files are maintained in REDCap at Northwestern University, a highly secure web-based research database. This database is used to schedule and track study visits; it is completely password protected. The tracking data base stores contact preferences; all communications strictly follow the participants' contact limitations. Computer data files never have any identifying information, and are encrypted for transfer between

study sites. Data files do not include information that could be used to identify the participant from the data file alone.

Dimagi will provide a private cloud server for the SMS application content and user data for the duration of the application deployment. This is a secure system and will be further protected by login credentials for limited access, to protect participant confidentiality. To protect privacy and confidentiality, we will encourage participants to delete text messages after taking medication, to use confidential messages that do not reveal HIV status or mention medications, and we will provide each participant with a fact sheet about cell phone confidentiality (e.g., passcode protecting phone).

10.1.9.2 STUDY RECORDS RETENTION

Data files are exported from REDCap and imported into SPSS database for storage and analysis.

10.1.10 PROTOCOL DEVIATIONS

Deviations from this protocol will be reported

10.1.11 PUBLICATION AND DATA SHARING POLICY

This protocol will adhere to publications, and data and resource sharing policies of the PATC3H consortium and will be consistent with the relevant NIH policies, laws and regulations.

10.1.12 CONFLICT OF INTEREST POLICY

Study investigators at UI/UCH and Northwestern University will adhere to their institutional policies for reporting conflicts of interest.

10.2 ADDITIONAL CONSIDERATIONS

N/A

10.3 ABBREVIATIONS AND SPECIAL TERMS

As described in the protocol.

10.4 PROTOCOL AMENDMENT HISTORY

*. A **Summary of Changes** table for the current amendment is located in the **Protocol Title Page**.*

Version	Date	Description of Change	Brief Rationale

11 REFERENCES

.

REFERENCES

1. Turning the tide against AIDS will require more concentrated focus on adolescents and young people [press release]. UNICEF, September 2017.

2. National Agency for the Control of AIDS (NACA). *National HIV and AIDS Strategic Framework, 2017-2021.* 2017.

3. National Agency for the Control of AIDS (NACA). *Global AIDS response country progress report.* Abuja, Nigeria2015.

4. Nigerian Federal Ministry of Heath. *National HIV & AIDS and reproductive health survey 2012 (NARHS Plus II).* 2013.

5. UNAIDS: Joint United Nations Programme on HIV/AIDS. *2015 Progress Report on the Global Plan.* Geneva, Switzerland: UNAIDS: Jointed United Nations Programme on HIV/AIDS;2015.

6. Nigerian Federal Ministry of Heath. *Integrated behavioural and biological survey.* National HIV/AIDS & STIs Control Programme;2014.

7 Keshinro B, Crowell TA, Nowak RG, et al. High prevalence of HIV, chlamydia and gonorrhoea among men who have sex with men and transgender women attending trusted community centres in Abuja and Lagos, Nigeria. *J Int AIDS Soc.* 2016;19(1):21270.

8. Vu L, Adebajo S, Tun W, et al. High HIV prevalence among men who have sex with men in Nigeria: implications for combination prevention. *J Acquir Immune Defic Syndr.* 2013;63(2):221-227.

9. Nigerian Federal Ministry of Heath. *National HIV & AIDS and reproductive health survey 2010 (NARHS Plus II).* 2010.

10. Sheehy M, Tun W, Vu L, Adebajo S, Obianwu O, Karlyn A. High levels of bisexual behavior and factors associated with bisexual behavior among men having sex with men (MSM) in Nigeria. *AIDS Care.* 2014;26(1):116-122.

11. Volz EM, Ndembi N, Nowak R, et al. Phylodynamic analysis to inform prevention efforts in mixed HIV epidemics. *Virus Evol.* 2017;3(2):vex014.

12. Bamgboye EA, Badru T, Bamgboye A. Transactional Sex between Men and Its Implications on HIV and Sexually Transmitted Infections in Nigeria. *J Sex Transm Dis.* 2017;2017:1810346.

13. World Health Organization. *HIV and adolescents: Guidance for HIV testing and counselling and care for adolescents living with HIV.* Geneva, Switzerland: World Health Organization;2013.

14. MacPhail CL, Pettifor A, Coates T, Rees H. "You must do the test to know your status": attitudes to HIV voluntary counseling and testing for adolescents among South African youth and parents. *Health Educ Behav.* 2008;35(1):87-104.

15. Kurth AE, Lally MA, Choko AT, Inwani IW, Fortenberry JD. HIV testing and linkage to services for youth. *J Int AIDS Soc.* 2015;18(2 Suppl 1):19433.

16. Brenhouse HC, Andersen SL. Developmental trajectories during adolescence in males and females: a cross-species understanding of underlying brain changes. *Neurosci Biobehav Rev.* 2011;35(8):1687-1703.

17. Mills KL, Goddings AL, Clasen LS, Giedd JN, Blakemore SJ. The developmental mismatch in structural brain maturation during adolescence. *Dev Neurosci.* 2014;36(3-4):147-160.

18. World Health Organization. *Consolidated guidelines on the use of antiretroviral drugs for treatment and preventing HIV infections: What's new.* Geneva, Switzerland: World Health Organization;2015.

19. Kanters S, Park JJ, Chan K, et al. Interventions to improve adherence to antiretroviral therapy: a systematic review and network meta-analysis. *Lancet HIV.* 2017;4(1):e31-e40.

20. World Health Organization. ADOLESCENT HIV TESTING, COUNSELLING AND CARE: Implementation guidance for health providers and planners. 2017. Accessed December 1, 2017.

21. World Health Organization. *The adolescent with a chronic condition: Epidemiology, developmental issues abnd health care provision.* Geneva, Switzerland: World Health Organization;2007.

22. Bennett GG, Glasgow RE. The delivery of public health interventions via the Internet: actualizing their potential. *Annu Rev Public Health.* 2009;30:273-292.

23. Catalani C, Philbrick W, Fraser H, Mechael P, Israelski DM. mHealth for HIV Treatment & Prevention: A Systematic Review of the Literature. *Open AIDS J.* 2013;7:17-41.

24. Devi BR, Syed-Abdul S, Kumar A, et al. mHealth: An updated systematic review with a focus on HIV/AIDS and tuberculosis long term management using mobile phones. *Comput Methods Programs Biomed.* 2015;122(2):257-265.

25. Howard AA, Hirsch-Moverman Y, Frederix K, et al. The START Study to evaluate the effectiveness of a combination intervention package to enhance antiretroviral therapy uptake and retention during TB treatment among TB/HIV patients in Lesotho: rationale and design of a mixed-methods, cluster-randomized trial. *Glob Health Action.* 2016;9(1):31543.

26. Hirsch-Moverman Y, Daftary A, Yuengling KA, et al. Using mHealth for HIV/TB Treatment Support in Lesotho: Enhancing Patient-Provider Communication in the START Study. *J Acquir Immune Defic Syndr.* 2017;74 Suppl 1:S37-S43.

27. Lester RT, Ritvo P, Mills EJ, et al. Effects of a mobile phone short message service on antiretroviral treatment adherence in Kenya (WelTel Kenya1): a randomised trial. *Lancet.* 2010;376(9755):1838-1845.

28. Graham SM, Micheni M, Kombo B, et al. Development and pilot testing of an intervention to promote care engagement and adherence among HIV-positive Kenyan MSM. *AIDS.* 2015;29 Suppl 3:S241-249.

29. NCC board and management in a strategic retreat [press release]. Abuja, Nigeria: Nigerian Communications Commission, October 7 2017.

30. Africapractice. *The social media landscape in Nigeria.* Nigeria: Africapractice;2014.

31. Cao B, Gupta S, Wang J, et al. Social Media Interventions to Promote HIV Testing, Linkage, Adherence, and Retention: Systematic Review and Meta-Analysis. *J Med Internet Res.* 2017;19(11):e394.

32. Tanner AE, Mann L, Song E, et al. weCARE: A Social Media-Based Intervention Designed to Increase HIV Care Linkage, Retention, and Health Outcomes for Racially and Ethnically Diverse Young MSM. *AIDS Educ Prev.* 2016;28(3):216-230.

33. Ko NY, Hsieh CH, Wang MC, et al. Effects of Internet popular opinion leaders (iPOL) among Internet-using men who have sex with men. *J Med Internet Res.* 2013;15(2):e40.

34. Young SD, Cumberland WG, Nianogo R, Menacho LA, Galea JT, Coates T. The HOPE social media intervention for global HIV prevention in Peru: a cluster randomised controlled trial. *Lancet HIV.* 2015;2(1):e27-32.

35. Taiwo BO, Idoko JA, Welty LJ, et al. Assessing the viorologic and adherence benefits of patient-selected HIV treatment partners in a resource-limited setting. *J Acquir Immune Defic Syndr.* 2010;54(1):85-92.

36. Adetunji AA, Muyibi SA, Imhansoloeva M, et al. Mobile phone use for a social strategy to improve antiretroviral refill experience at a low-resource HIV clinic: patient responses from Nigeria. *AIDS Care.* 2017;29(5):575-578.

37. Garofalo R, Kuhns LM, Hotton A, Johnson A, Muldoon A, Rice D. A Randomized Controlled Trial of Personalized Text Message Reminders to Promote Medication Adherence Among HIV-Positive Adolescents and Young Adults. *AIDS Behav.* 2016;20(5):1049-1059.

38. Steward WT, et al. Peer navigation enhances HIV care retention: an RCT in South African primary clinics. Conference on Retrorivuses and Opportunistic Infections (CROI); 2017; Seattle, WA.

39. Steward WT, Sumitani J, Moran ME, et al. Engaging HIV-positive clients in care: acceptability and mechanisms of action of a peer navigation program in South Africa. *AIDS Care.* 2017:1-8.

40. Lippman SA, Shade SB, Sumitani J, et al. Evaluation of short message service and peer navigation to improve engagement in HIV care in South Africa: study protocol for a three-arm cluster randomized controlled trial. *Trials.* 2016;17:68.

41. Linnemayr S, Huang H, Luoto J, et al. Text Messaging for Improving Antiretroviral Therapy Adherence: No Effects After 1 Year in a Randomized Controlled Trial Among Adolescents and Young Adults. *Am J Public Health.* 2017;107(12):1944-1950.

42 Gandhi M, Ameli N, Bacchetti P, et al. Atazanavir concentration in hair is the strongest predictor of outcomes on antiretroviral therapy. *Clin Infect Dis.* 2011;52(10):1267-1275.

43. Gandhi M, Ameli N, Bacchetti P, et al. Protease inhibitor levels in hair strongly predict virologic response to treatment. *AIDS.* 2009;23(4):471-478.

44. van Zyl GU, van Mens TE, McIlleron H, et al. Low lopinavir plasma or hair concentrations explain second-line protease inhibitor failures in a resource-limited setting. *J Acquir Immune Defic Syndr.* 2011;56(4):333-339.

45. Baxi SM, Greenblatt RM, Bacchetti P, et al. Nevirapine Concentration in Hair Samples Is a Strong Predictor of Virologic Suppression in a Prospective Cohort of HIV-Infected Patients. *PLoS One.* 2015;10(6):e0129100.

46. Belzer ME, Naar-King S, Olson J, et al. The use of cell phone support for non-adherent HIV-infected youth and young adults: an initial randomized and controlled intervention trial. *AIDS and behavior.* 2014;18(4):686-696.

47. Naar-King S, Templin T, Wright K, Frey M, Parsons J, Lam P. Psychosocial factors and medication adherence in HIV-positive youth. *AIDS Patient Care and STDs.* 2006;20 (1):44-47.

48. Balfour L, Kowal J, Tasca G, et al. Development and psychometric validation of the HIV treatment knowledge scale. *AIDS Care.* 2007;19(9):1141-1148.

49. Erlen JA, Cha ES, Kim KH, Caruthers D, Sereika SM. The HIV Medication Taking Self-efficacy Scale: psychometric evaluation. *Journal of advanced nursing.* 2010;66(11):2560-2572.

50. Wright K, Naar-King S, Lam P, Templin T, Frey M. Stigma scale revised: Reliability and validity of a brief measure of stigma for HIV + youth. *Journal of Adolescent Health.* 2008;40(1):96-98.

51. Kolbum K, Prencipe L, Hjelm L, Peterman A, Handa S, Palemo T. Examination of performance of the center for epidemiologic studies depression scale short form 10 among african youth in poor, rural households. *BMC Psychiatry.* 2018;18:201.

52. Humeniuk R. *Validation of the alcohol, smoking and substance involvement screening test (ASSIST) and pilot brief intervention: A technical report of phase II findings of the WHO ASSIST project.* Geneva: World Health Organization;2006.

53. Larsen DL, Attkisson CC, Hargreaves WA, Nguyen TD. Assessment of client/patient satisfaction: Development of a general scale. *Evaluation and Program Planning.* 1979;2:197-207.

54. Larsen DL, Attkisson CC, Hargreaves WA, Nguyen TD. Assessment of client/patient satisfaction: Development of a general scale. *Evaluation and Program Planning.* 1979;2:197-207.
