# Supplementary material for: Evaluation of the iCARE Nigeria Pilot Intervention Using Social Media and Peer Navigation to Promote HIV Testing and Linkage to Care Among High-Risk Young Men: A Nonrandomized Controlled Trial
Source: JAMA Netw Open. 2022 Feb 22;5(2):e220148. doi: 10.1001/jamanetworkopen.2022.0148 (PMC8864509; doi:10.1001/jamanetworkopen.2022.0148)
Supplement: Supplement 2. — Data Sharing Statement [file jamanetwopen-e220148-s002.pdf]

Data Sharing Statement

Garofalo. Evaluation of the iCARE Nigeria Pilot Intervention Using Social Media and Peer Navigation to Promote HIV Testing and Linkage to Care Among High-Risk Young Men. *JAMA Netw Open*. Published February 22, 2022. doi:10.1001/jamanetworkopen.2022.0148

Data
Data available: Yes
Data types: Deidentified participant data
How to access data: lkuhns@luriechildrens.org -- Lisa Kuhns
When available: With publication

Supporting Documents
Document types: Statistical/analytic code, Informed consent form
How to access documents: Lisa Kuhns- lkuhns@luriechildrens.org
When available: With publication

Additional Information
Who can access the data: Anyone requesting data
Types of analyses: Any purposes
Mechanisms of data availability: With support
Any additional restrictions: None
